# Marine fish parasites of Vietnam: a comprehensive review and updated list of species, hosts, and zoogeographical distribution[Fn FN1]

**DOI:** 10.1051/parasite/2022033

**Published:** 2022-07-14

**Authors:** Van Thuong Truong, Huong Thi Thuy Ngo, Te Quang Bui, Harry W. Palm, Rodney A. Bray

**Affiliations:** 1 Aquaculture and Sea-Ranching, Faculty of Agricultural and Environmental Sciences, University of Rostock Justus-von-Liebig-Weg 6 18059 Rostock Germany; 2 Fisheries and Technical Economic College, Dinh Bang 16315 Tu Son, Bac Ninh Vietnam; 3 Faculty of Biotechnology, Chemistry and Environmental Engineering, Phenikaa University Hanoi 12116 Vietnam; Bioresource Center, Phenikaa University Hanoi 12116 Vietnam; 4 Research Institute for Aquaculture No. 1, Dinh Bang 16315 Tu Son, Bac Ninh Vietnam; 5 Department of Life Sciences, Natural History Museum Cromwell Road London SW7 5BD United Kingdom

**Keywords:** Marine fish parasites, Species richness, Diversity, Vietnam, Host, Distribution

## Abstract

With a long coastline stretching from tropical to subtropical climate zones, and an immense exclusive economic zone with over 4000 islands, the Vietnamese marine waters support a rich and biodiverse parasite fauna. Although the first parasitological record was in 1898, systematic studies of the parasite fauna have increased during the last 50 years. This comprehensive review covers the current state of knowledge of marine fish parasites in Vietnam and lists 498 species found in 225 fish species, and their geographical distribution. In addition, 251 marine parasite species have newly been added to the already known fauna of 247 species since 2006 (more than two-fold increase). The most speciose group was the Digenea, which accounted for 43% of the total parasite species biodiversity, followed by Monogenea (23.5%), Crustacea (11.6%), Nematoda, and Acanthocephala (8.0% each). The shallow and muddy Gulf of Tonkin showed a rich parasite fauna, accounting for 66.3% of the whole marine parasite fauna of Vietnam, with Digenea accounting for 51% of the regional total parasite richness, followed by Monogenea (27%), Acanthocephala (8.8%), and Nematoda (5.8%). Only a few species belonged to Hirudinea, Myxozoa, and Cestoda, suggesting that these taxa may be understudied. Despite significant progress in studies of marine fish parasites in Vietnam since 2006, only about 12% and 13% of the total fish species have been examined for parasites in the whole country and the Gulf of Tonkin, respectively.

## Abstract (fr)

Avec un long littoral s’étendant des zones climatiques tropicales à subtropicales et une immense zone économique exclusive incluant plus de 4 000 îles, les eaux marines vietnamiennes abritent une faune parasitaire abondante et riche en biodiversité. Le premier signalement parasitologique remonte à 1898 et les études systématiques de la faune parasitaire se sont multipliées au cours des 50 dernières années. Cette synthèse complète couvre l’état actuel des connaissances sur les parasites des poissons marins au Vietnam et répertorie 498 espèces trouvées dans 225 espèces de poissons et leur répartition géographique. De plus, 251 espèces de parasites marins ont été nouvellement ajoutées à la faune déjà connue de 247 espèces depuis 2006 (soit une augmentation de plus du double). Le groupe le plus riche en espèces était les Digenea, qui représentaient 43% de la biodiversité totale des espèces de parasites, suivis des Monogenea (23,5 %), des Crustacea (11,6 %), des Nematoda et des Acanthocephala (8,0 % chacun). Le golfe peu profond et boueux du Tonkin a montré une riche faune parasitaire, représentant 66,3 % de l’ensemble de la faune parasitaire marine du Vietnam, avec les Digenea représentant 51 % de la richesse parasitaire totale régionale, suivi des Monogenea (27 %), Acanthocephala (8,8 %) et Nematoda (5,8 %). Seules quelques espèces appartenaient aux Hirudinea, Myxozoa et Cestoda, ce qui suggère que ces taxons pourraient être sous-étudiés. Malgré des progrès significatifs dans les études sur les parasites des poissons marins au Vietnam depuis 2006, seulement 12 % et 13 % des espèces de poissons ont été examinées, respectivement pour les parasites de l’ensemble du pays et du golfe du Tonkin.

## Introduction

Vietnam has a long coastline of more than 3260 km and an immense exclusive economic zone (one million km^2^), comprising the coastlines of more than 4000 islands and thousands of square kilometers of coral reefs [120, 131]. The Vietnamese Sea stretches from tropical to subtropical climate zones (from the Namzu Islands, 9°40′ N, 104°22′ E, in the Gulf of Thailand to Daochao Island, 20°50′ N, 107°20′ E, in the Gulf of Tonkin), opening to the Pacific Ocean and being known for its biodiverse marine ecosystems supporting a rich fauna and flora, including fish parasites. Although Billet [26] recorded the first parasite, *Distomum hypselobagri* (Trematoda), systematic studies of the parasite fauna did not begin until the 1960s (e.g., [69, 71, 82, 97–99], etc.), and the first review was published only 16 years ago [19]. Recently, studies using both molecular [11–13, 16, 20, 24, 25, 36] and morphological methods [12, 13, 20, 24, 92] have been conducted in order to elucidate the systematics of marine parasites in Vietnam.

According to Palm and Bray [103], marine fish parasites are important in ecosystems. In open Hawaiian waters (Pacific Ocean), the authors discovered an average of 2.2 parasite species per marine fish species. In contrast, Klimpel et al. [66] discovered 1.5 metazoan parasite species per deep-sea fish species. According to Palm [102], each fish species in the world harbors up to 3–4 metazoan parasite species on average. In this sense, Arthur and Te [19] reported that the estimated number of parasite species per marine fish species in Vietnamese waters was as high as 3.0. There are currently 1876 species of marine fish reported in Vietnam’s marine waters [45]. Despite this, only 247 parasite species infecting 82 marine fish species were documented by 2006, accounting for about 4.4% of the total fish community [19]. Such data demonstrate that the number of parasites known to science is insufficient to provide credible estimates of the local parasite community.

Because of the importance of marine parasites in fisheries, aquaculture, and human health, more research has been conducted recently, focusing on commercially important fish (e.g., [88, 90, 132, 135]). Furthermore, because parasites can be used as a tool for monitoring climate change and environmental health [86, 102, 129], studies regarding these organisms are essential. However, no systematic update pertaining to parasites in marine fish from Vietnam has been published since 2006, and since that date, much has changed in the knowledge of such organisms. However, Poulin [123] asserts that, due to the absence of an adequate method for determining parasite diversity, species richness remains the most straightforward and thus most relevant indicator of diversity. Therefore, the present study is aimed at: (1) shedding more light on the marine parasite richness in Vietnam by listing, correcting, and arranging the latest information, including the host and geographical distribution of parasites of marine fish; and (2) providing a current state of knowledge on parasite research in the Vietnamese marine waters, with particular emphasis on the Gulf of Tonkin (GOT).

## Methods

### Study sites and data collection

Vietnam is a Southeast Asian country divided into three distinct geographical and climatic regions: North, Central, and South Vietnam. The aquaculture industry is vital to the Vietnamese economy, especially brackish and marine fish farming along the country’s coastline, particularly in the north [67].

A total of 137 references published between 1901 and 2022 were retrieved to gather knowledge on marine fish parasites and their occurrence in cultured and wild marine fish in Vietnam, focusing on the latest studies. This list was also based on information from the checklists of parasites of fishes in Vietnam [19], and it was expanded to provide a parasite-host list organized on a taxonomic basis, including information about each parasite species, sites of infection, the known geographical distribution in Vietnam, and references. More recently, reviews of individual species of parasites, including morphology, host-parasite distribution, zoogeography, and occasionally molecular analysis have become available; for instance, Amin et al. [2, 8, 14, 15] on *Rhadinorhynchus trachuri* Harada, 1935, *Neoechinorhynchus johnii* Yamaguti, 1939, *Sclerocollum neorubrimaris* Amin, Heckmann, Ha, 2018, respectively and *Serrasentis sagittifer* (Linton, 1889) Linton, 1932. The animal science databases (https://www.cabi.org/animalscience/disease-health/), MEDLINE, PubMed, Scopus, Web of Science, and Google Scholar were searched using the keywords: parasite, fish, marine, and Vietnam.

In addition, we included our most recent results from the Gulf of Tonkin, which included 40 *Acanthopagrus latus*, 1 *Acanthocybium solandri*, 7 *Neotrygon kuhlii*, 47 *Protonibea diacanthus*, 37 *Trachinotus blochii*, 7 *Telatrygon zugei*, and 2 *Thynnus* sp. (from 2014 to 2015). The fish were collected in the local fish markets in Ha Long (20°94′96″ N, 107°08′23″ E) and Cat Ba (20°72′75″ N, 107°04′67″ E) between 2014 and 2015, kept on ice and transferred to the Fisheries College’s laboratory in Bac Ninh (close to Ha Noi). The parasite examination was performed using a Nikon SMZ-1 stereomicroscope and the standard procedures described by Palm [101] and Palm and Bray [103]. For taxonomic identification, permanent mounts were prepared following standard methods for Digenea, Monogenea, Nematoda, Cestoda, Acanthocephala, and Crustacea (Isopoda, Copepoda) according to Riemann [124], Palm [101], Arai et al. [18], Dojiri and Ho [42] and Paladini et al. [100]. The method to identify trichodinid ciliates was modified after Klein [65]. Parasites were identified using an Olympus BX53F DIC microscope based on taxonomic keys and original descriptions. The monogenean identification followed Chisholm and Whittington [39], Dang et al. [41], and Hendrix [51]. The identification of crustaceans was based on Kabata and Margolis [59], Shultz [127], and Dojiri and Ho [42]. The digenean identification followed Bray et al. [32], Gibson et al. [47], Jones et al. [57], and Bray and Justine [30]. The identification of cestodes was based on Khalil [63] and Palm [101]. Identification of nematodes was based on Anderson et al. [17] and Gibbons [46]. The protozoan identification followed Bruno et al. [33], and Lom and Dyková [78]. Identification of acanthocephalans was based on Amin [1] and Arai et al. [18]. Other groups of invertebrates retrieved from the literature were identified using methods developed by different authors.

Furthermore, numerous scientific names for recorded hosts from the literature have been amended and corrected following the FishBase database (https://www.fishbase.in/search.php; 2021). The scientific names of existing parasites were carefully checked and corrected using various reliable sources, such as the World Register of Marine Species database (http://www.marinespecies.org/index.php), Ocean Biodiversity Information System (https://obis.org/), and Integrated Taxonomic Information System (https://www.itis.gov/).

Vietnam’s 3260 km of coastline is home to 25 coastal provinces and three cities by the sea. The following abbreviations are used to denote administrative and oceanic divisions where the parasites have been reported (Table 1)

**Table 1:** The abbreviated names of the municipalities, provinces, and sea or ocean parts where samples were collected.

Municipalities/provinces/sea or ocean parts		Municipalities/provinces/sea or ocean parts	
Full names	Abbreviation	Full names	Abbreviation
Bac Lieu province	BL	Nam Dinh province	ND
Binh Thuan province	BT	Nghe An province	NA
Gulf of Tonkin	GOT	Quang Binh province	QB
Gulf of Thailand	GOTh	Quang Ninh province	QN
Hai Phong city	HP	South China Sea	SCS
Ha Long Bay, Quang Ninh	HL-QN	Thanh Hoa province	TH
Thua Thien Hue province	Hue	Ba ria-Vung tau province	VT
Khanh Hoa province	KH		
Kien Giang province	KG	The Gulf of Tonkin (GOT) includes HP, NA, ND, QB, QN, and TH	

### Data analysis

The retrieved literature data, such as the reference, study period, study location, site of infection, fish host, taxa, and parasite species studied, were entered into an excel spreadsheet. This analysis only included parasites identified at the species level. In the list, the parasite taxon levels, i.e., subfamilies, genera, and species, were organized alphabetically. For the entire country of Vietnam and the Gulf of Tonkin, parasite species were classified into eight main taxa: Myxozoa (My), Ciliophora (Ci), Monogenea (Mo), Digenea (D), Cestoda (C), Nematoda (N), Acanthocephala (A), and Crustacea (Cr).

In order to have an insight into parasite richness, the total number of parasite species for Vietnam and the GOT was used to calculate the parasite species ratio per fish host. It is determined by dividing the total number of parasite species parasitizing fishes by the number of infected hosts. Microsoft Excel software was used to perform calculations and descriptive analysis of the collected data.

## Results and discussion

### Parasites in marine fish of Vietnam

Table 2 contains information about the parasite and host taxa, the site of infection, geographical localities, and literature sources.

**Table 2:** List of marine fish parasites in Vietnamese waters.

Parasite		Taxa			Hosts		Site of infection	Locality/ies		References	
**Phylum: Myzozoa Cavalier-Smith & Chao, 2004**											
**Class: Conoidasida Levine, 1988**											
**Order: Eucoccidiorida Léger, 1911**											
**Family: Eimeriidae Minchin, 1903**											
*Goussia* sp.		My			*Lates calcarifer*		Sw	KH		[122]	
**Phylum: Cnidaria Hatschek, 1888**											
**Class: Myxozoa Grassé, 1970**											
**Subclass: Myxosporea Bütschli, 1881**											
**Order: Bivavulida Shulman, 1959**											
**Family: Ceratomyxidea Doflein, 1899**											
*Ceratomyxa binhthuanensis* Chinh, Ha, Doanh, Violettta		My			*Epinephelus fasciatus*		Gb	KH		[38]	
*Ceratomyxa* sp.		My			*Epinephelus bleekeri*, *E. coioides*, *E. malabaricus*, *E. tauvina*, *Lates calcarifer*		Gb	KH, HP, ND		[122, 133, 135]	
**Family: Meglitschiidae Kovaleva, 1988**											
*Meglitschia insolita* (Meglitsch, 1960)		My			*Epinephelus coioides*		Gb	KH		[135]	
**Family: Myxobolidae Thélohan, 1892**											
*Henneguya cerebralis* Pronin, 1972		My			*Lates calcarifer*		Gi	KH		[122]	
*Henneguya lata* Nguyen, Chinh, Ngo, Van Tuc, Itoh, Yoshinaga, Shirakashi & Doanh, 2021 in [Chinh NN et al. (2021)]^1^		My			*Acanthopagrus latus*		Gi	QN		[37]	
*Henneguya* sp.		My			*Acanthopagrus latus*		Gi, Sw	QN		Present study	
*Myxobolus* sp.		My			*Epinephelus bleekeri*, *E. coioides*, *E. tauvina*		K	KH		[135]	
**Phylum: Ciliophora Doflein, 1901**											
**Class: Oligohymenophorea, Stein 1859**											
**Subclass: Peritrichia Stein, 1859**											
**Order: Mobilida Kahl, 1933**											
**Family: Trichodinidae Claus, 1951**											
*Trichodina japonica* Imai, Miyazaki & Nomura, 1991		Ci			*Epinephelus bleekeri*, *E. coioides*, *E. malabaricus*, *E. tauvina*		Gi	KH		[140]	
*Trichodina rostrata* Kulemina, 1968^1^		Ci			*Lates calcarifer*		Gi, Sk	KH		[122]	
*Trichodina* sp.		Ci			*Epinephelus bruneus*, *E. tauvina*, *E. sexfasciatus*, *E. coioides*, *Lutjanus erythropterus*, *Rachycentron canadum*, *Sciaenops ocellatus*		Gi, Sk	HP, QN, NA, KH, VT		[34, 121, 133, 140]	
**Order: Sessilida Stein 1933**											
**Family: Epistylididae Kahl, 1935**											
*Apiosoma* sp.		Ci			*Epinephelus bleekeri*, *E. coioides*, *E. malabaricus*, *E. tauvina*		Sk	KH		[135]	
**Family: Scyphiddidae Kahl, 1935**											
*Ambiphrya* sp.		Ci			*Epinephelus bleekeri*, *E. coioides*, *E. malabaricus*, *Lates calcarifer*		Sk	KH		[140]	
**Class: Phyllopharyngea de Puytorac, Batisse, Bohatier, Corliss, Deroux, Didier, Dragesco, Fryd-Versavel, Grain, Grollere, Horasse, Mode, Laval, Roque, Savoie & Tuffrau, 1974**											
**Order: Chlamydodontida Deroux, 1976**											
**Family: Chilodinellidae Deroux, 1970**											
*Chilodonella* sp.		Ci			*Epinephelus bleekeri*, *E. coioides*		Sk	KH		[135]	
**Order: Dysteriida Deroux, 1970**											
**Family: Hartmannulidae Poche, 1913**											
*Brooklynella hostilis* Lom & Nigrelli, 1970		Ci			*Epinephelus bleekeri*, *E. bruneus*, *E. sexfasciatus*, E. tauvina*		Gi, Sk	QN, KH		[34, 140]	
**Class: Prostomatea Schewiakoff, 1896**											
**Order: Prorodontida Corliss, 1974**											
**Family: Holophryidae Perty, 1852**											
*Cryptocaryon irritans* Brown, 1951		Ci			*Epinephelus bleekeri*, *E. coioides*, *E. malabaricus*, *Lates calcarifer*, *Sciaenops ocellatus*		Gi, Sk	KH, HP, ND		[133, 140]	
**Phylum: Platyhelminthes Minot, 1876**											
**Class: Monogenea Van Beneden, 1858**											
**Subclass: Monopisthocotylea Odhner, 1912**											
**Order: Capsalidea Lebedev, 1988**											
**Family: Capsalidae Baird, 1853**											
*Allobenedenia epinepheli* (Bychowsky & Nagibina, 1967) Yang, Kritsky & Sun, 2004	Mo			*Epinephelus coioides*			Gi	KH		[140]	
*Allobenedenia yamagutii* (Egorova, 1994) Yang, Kritsky & Sun, 2004	Mo			*Hyporthodus nigritus* (Holbrook, 1855)			Gi, Sk	KH, QN		[88, 140]	
*Benedenia epinepheli* (Yamaguti, 1937) Meserve, 1938	Mo			*Epinephelus bleekeri*, *E. bruneus*, *E. coioides*, *E. malabaricus*, *E. sexfasciatus*, E. tauvina*, *Lutjanus argentimaculatus*			Sk, Fi	KH, QN		[34, 135]	
*Benedenia* sp.	Mo			*Epinephelus bruneus*, *E. coioides*, *E. sexfasciatus*, *E. tauvina* *, *Lutjanus erythropterus*, *Rachycentron canadum*			Sk	HP, ND, QN, KH		[34, 133, 140]	
*Capsala affinis* (Mamaev, 1968) Chisholm & Whittington, 2007	Mo			*Auxis thazard*, *Euthynnus affinis*			Gi	SCS		[80]	
*Capsala notosinense* (Mamaev, 1968) Chisholm & Whittington, 2007	Mo			*Euthynnus affinis*			Gi	SCS		[80]	
*Capsala paucispinosa* (Mamaev, 1968) Chisholm & Whittington, 2007	Mo			*Euthynnus affinis, Thunnus thynnus*			Gi, Sk	SCS		[80]	
*Capsala* sp.	Mo			*Thunnus thynnus*			Gi	SCS		[80]	
*Encotyllabe spari* Yamaguti, 1934	Mo			*Gymnocranius griseus*, *Plectorhinchus* sp.			Gi	GOT		[81]	
*Megalocotyle lutiani* Lebedev, 1970	Mo			*Lutjanus lutjanus*			Gi	GOT		[73]	
*Neobenedenia melleni* (MacCallum, 1927) Yamaguti, 1963	Mo			*Epinephelus bleekeri*, *E. coioides*, *E. malabaricus*, *Lates calcarifer*, *Lutjanus argentimaculatus*			Sk, Fi	KH		[53, 140]	
*Neobenedenia* sp.	Mo			*Rachycentron canadum*			Sk	KH		[53]	
*Sessilorbis limopharynx* Mamaev, 1970	Mo			*Platax orbicularis*			Gi	GOT		[81]	
*Sprostoniella multitestis* Bychowsky & Nagibina, 1967	Mo			*Platax orbicularis*			Gi	GOT		[81]	
*Trilobiodiscus lutiani* Bychowsky & Nagibina, 1967	Mo			*Lutjanus argentimaculatus*			–	GOT		[43]	
*Trochopus antigoniae* Egorova & Korotaeva, 1990		Mo			*Antigonia rubescens*		Gi	SCS		[44]	
**Order: Dactylogyridea Bychowsky, 1937**											
**Family: Ancyrocephalidae Bychowsky, 1937**											
*Ancyrocephalus macrogaster* Yamaguti, 1953		Mo			*Gerres filamentosus*		Gi	GOT		[81]	
*Ancyrocephalus parspinicirrus* Mamaev, 1970		Mo			*Drepane punctata*		Gi	GOT		[81]	
*Ancyrocephalus unicirrus* Tripathi, 1959		Mo			*Pomadasys argenteus*		Gi	GOT		[81]	
*Haliotrema bilobatum* (Yamaguti, 1953) Bychowsky & Nagibina, 1970		Mo			*Drepane longimana*, *D. punctata*		Gi	GOT		[81]	
*Haliotrema cromileptis* Young, 1968		Mo			*Epinephelus bleekeri*, *E. coioides*		Gi	KH		[41, 135]	
*Haliotrema epinepheli* Yamaguti, 1968		Mo			*Epinephelus bleekeri*, *E. coioides*		Gi	KH		[41, 135]	
*Haliotrema geminatohamula* Bychowsky & Nagibina, 1971		Mo			*Leiognathus nuchalis*		Gi	QN		[88]	
*Haliotrema spinicirrus* (Yamaguti, 1953) Bychowsky & Nagibina, 1970		Mo			*Decapterus maruadsi*, *Drepane punctata*		Gi	QN, GOT		[81, 88]	
*Haliotrema* sp.		Mo			*Acanthopagrus latus*, *Epinephelus bruneus*, *E. bleekeri*, *E. coioides*, *E. sexfasciatus*, E. tauvina*		Gi	GOT, KH		[34, 41, 135] Present study	
*Ligophorus hamulosus* Pan & Zhang in Pan 1999		Mo			*Moolgarda engeli*, *M. seheli*		Gi	QN		[88]	
*Metahaliotrema kulkarnii* Venkatanarasaiah, 1981		Mo			*Scatophagus argus*		Gi	GOT		[68]	
*Metahaliotrema mizellei* Venkatanarasaiah, 1981		Mo			*Scatophagus argus*		Gi	QN, GOTh		[68, 88]	
*Metahaliotrema scatophagi* Yamaguti, 1953		Mo			*Scatophagus argus*		Gi	QN		[68]	
*Metahaliotrema simile* Kritsky, Nguyen, Ha & Heckman, 2016		Mo			*Scatophagus argus*		Gi	QN		[68]	
*Metahaliotrema yamagutii* Mizelle & Price, 1964		Mo			*Scatophagus argus*		Gi	GOTh		[68]	
*Metahaliotrema ypsilocleithrum* Kritsky, Nguyen, Ha & Heckman, 2016		Mo			*Scatophagus argus*		Gi	QN, GOTh		[68]	
*Murraytrema pricei* Bychowsky & Nagibina, 1977		Mo			*Nibea albiflora*		Gi	QN		[88]	
*Paradiplectanotrema trachuri* (Kovaleva, 1970) Gerasev, Gayevskaya & Kovaleva, 1987		Mo			*Argyrosomus japonicus*, *Johnius carouna*		St	QN		[88]	
*Platycephalotrema platycephali* (Yin & Sproston, 1948) Kritsky & Nitta, 2019		Mo			*Platycephalus indicus*		Gi	QN		[88]	
*Protogyrodactylus scapulasser* (Mamaev, 1970) Gusev, 1973		Mo			*Gerres filamentosus*		Gi	GOT		[81]	
**Family: Diplectanidae Monticelli, 1903**											
*Calydiscoides flexuosus* (Yamaguti, 1953) Young, 1969		Mo			*Nemipterus Japonicus*		Gi	QN		[88]	
*Acleotrema nenue* (Yamaguti, 1968) Dominques & Boeger, 2007		Mo			*Lates calcarifer*		Gi, Sk	KH		[122]	
*Diplectanum* sp.		Mo			*Plectorhinchus cinctus*		Gi	GOT		[81]	
*Laticola latesi* (Tripathi, 1959) Yang, Kritsky, Sun, Zhang, Shi & Agrawal, 2006		Mo			*Lates calcarifer*		Gi, Sk	KH		[122]	
*Laticola paralatesi* (Nagibina, 1976) Yang, Kritsky, Sun, Zhang, Shi & Agrawal, 2006		Mo			*Lates calcarifer*		Gi, Sk	KH		[122]	
*Murraytrema pricei* Bychowsky & Nagibina, 1977		Mo			*Nibea albiflora*		Gi	QN		[88]	
*Paradiplectanum blairense* (Gupta & Khanna, 1974) Domingues & Boeger, 2008		Mo			*Sillago japonica*, *S. sihama*		Gi	QN		[88]	
*Pseudorhabdosynochus coioidesis* Bu, Leong, Wong, Woo & Foo, 1999		Mo			*Epinephelus bleekeri*, *E. coioides*, *E. malabaricus*, *E. tauvina*		Gi	KH		[135]	
*Pseudorhabdosynochus cupatus* (Young, 1969) Kritsky & Beverley-Burton, 1986		Mo			*Epinephelus sexfasciatus*		Gi	QN		[34]	
*Pseudorhabdosynochus epinepheli* (Yamaguti, 1938) Kritsky & Beverley-Burton, 1986		Mo			*Epinephelus bleekeri*, *E. bruneus*, *E. coioides*, *E. sexfasciatus*, *E. tauvina*		Gi	QN, KH		[34, 135]	
*Pseudorhabdosynochus grouperi* (Bu, Leong, Wong, Woo & Foo, 1999) Wu, Li, Zhu & Xie, 2005		Mo			*Epinephelus bleekeri*, *E. coioides*, *E. malabaricus*, *E. tauvina*, *Lates calcarifer*		Gi	KH		[140]	
*Pseudorhabdosynochus lantauensis* (Beverley-Burton & Suriano, 1981) Kritsky & Beverley-Burton, 1986		Mo			*Epinephelus bleekeri*, *E. coioides*, *E. malabaricus*		Gi	KH		[135]	
*Pseudorhabdosynochus serrani* (Yamaguti, 1953) Kritsky & Beverley-Burton, 1986		Mo			*Epinephelus bleekeri*, *E. coioides*, *E. malabaricus*, *E. tauvina*		Gi	KH		[135]	
*Pseudorhabdosynochus summanoides* Yang, Gibson & Zeng, 2005		Mo			*Epinephelus bleekeri*, *E. coioides*, *E. malabaricus*		Gi	KH		[135]	
*Pseudorhabdosynochus summanae* (Young, 1969) Kritsky & Beverley-Burton, 1986		Mo			*Epinephelus coioides*, *E. bleekeri*		Gi	KH		[135]	
*Pseudorhabdosynochus* sp.		Mo			*Epinephelus coioides*		Gi	QN, HP, ND		[133]	
**Order: Gyrodactylidea Price, 1943**											
**Family: Tetraonchoididae Bychowsky, 1951**											
*Paratetraonchoides inermis* Bychowsky, Gussev & Nagibina, 1965		Mo			*Ichthyscopus lebeck*		Gi	GOT		[35]	
*Pavlovskioides ichthyoscopi* Bychowsky, Gussev & Nagibina, 1965		Mo			*Ichthyscopus lebeck*		Gi	GOT		[35]	
*Pavlovskioides litoralis* Bychowsky, Gussev & Nagibina, 1965		Mo			*Trachinocephalus myops*		Gi	GOT		[35]	
*Pseudotetraonchoides bleekeriae* Bychowsky, Gussev & Nagibina, 1965		Mo			*Bleekeria viridianguilla*		Gi	GOT		[35]	
**Subclass: Polyopisthocotylea Odhner, 1912**											
**Order: Mazocraeidea Bychowsky, 1957**											
**Family: Allodiscocotylidae Tripathi, 1959**											
*Allodiscocotyla chorinemi* Yamaguti, 1953		Mo			*Caranx* sp., *Decapterus* sp., *Scomberoides lysan*		Gi	GOT, SCS		[73, 76, 118]	
*Allodiscocotyla diacanthi* Unnithan, 1962		Mo			*Acanthopagrus pacificus*, *Decapterus* sp.		Gi	QN, GOT, SCS		[73, 76, 93, 118]	
*Camopia rachycentri* Lebedev, 1970		Mo			*Rachycentron canadum*		Gi	GOT		[74]	
*Metacamopia chorinemi* (Yamaguti, 1953) Lebedev, 1984		Mo			*Scomberoides lysan*, *Selar crumenophthalmus*		Gi	GOT, SCS		[73, 76, 118]	
*Metacamopia lebedevi* Nguyen, Nguyen, Ha, Ngoc, Ngoc, Le, Tatonova & Greiman, 2020		Mo			*Acanthopagrus pacificus*		Gi	QN		[93]	
**Family: Axinidae Monticelli, 1903**											
*Allopseudaxine macrova* (Unnithan, 1957) Yamaguti, 1963		Mo			*Auxis thazard*, *Caranx* sp.		Gi	SCS		[72, 80]	
*Loxuroides pricei* Nguyen, Nguyen, Bui & Ha, 2016		Mo			*Cypselurus naresii*		Gi	GOT, QB		[92]	
*Unnithanaxine naresii* Nguyen, Nguyen, Bui & Ha, 2016		Mo			*Cypselurus naresii*		Gi	GOT, Hue		[92]	
**Family: Bychowskicotylidae Lebedev, 1969**											
*Bychowskicotyle plectorhynchi* Lebedev, 1969		Mo			*Plectorhinchus cinctus*		Gi	GOT		[74]	
*Yamaguticotyla jucunda* (Lebedev, 1972) Lebedev, 1984		Mo			Pomadasyidae gen. sp.		Gi	GOT		[74]	
**Family: Diclidophoridae Cerfontaine, 1895**											
*Osphyobothrus bychowskyi* Khoche & Chauhan, 1969		Mo			*Saurida tumbil*		Gi	GOT, SCS		[83, 114]	
**Family: Gastrocotylidae Price, 1943**											
*Churavera triangula* (Mamaev, 1967) Lebedev, 1986		Mo			*Auxis thazard*		Gi	SCS		[74, 80]	
*Engraulicola thrissocles* (Tripathi, 1959) Lebedev, 1971		Mo			*Anchoviella sp*., *Thryssa mystax*		Gi	GOT		[74]	
*Gastrocotyle indica* Subhapradha, 1951		Mo			*Alepes djedaba*			SCS		[74]	
*Gastrocotyle kurra* Unnithan, 1968		Mo			*Decapterus* sp.		Gi	GOT		[74]	
*Gastrocotyle trachuri* Van Beneden & Hesse, 1863		Mo			*Decapterus* sp., *Selar crumenophthalmus*, *Trachurus trachurus*		Gi	GOT, SCS		[73, 76, 118]	
*Gastrocotyle* sp.		Mo			*Alectis indicus*, *Decapterus* sp.		Gi	GOT, SCS		[73, 76, 118]	
*Pseudaxine bivaginalis* Dillon & Hargis, 1965		Mo			*Selaroides leptolepis*		Gi	GOT		[72]	
*Pseudaxine trachuri* Parona & Perugia, 1890		Mo			*Carangoides malabaricus*, *Caranx* sp., *Decapterus* sp.		Gi	GOT, SCS		[73, 76, 118]	
*Pseudaxine bychowskyi* (Lebedev, 1977) Bouguerche, Tazerouti, Gey & Justine, 2020		Mo			*Alepes djedaba*, *Alepes kleinii*		Gi	SCS		[74]	
*Pseudaxine caballeroi* (Lebedev, 1977) Bouguerche, Tazerouti, Gey & Justine, 2020		Mo			*Alepes djedaba*, *Alepes kleinii*		Gi	SCS		[74]	
*Pseudaxine* sp.		Mo			*Alectis indicus*, *Auxis thazard*, *Caranx* sp., *D. muroadsi*, *Decapterus* sp*.*		Gi	QN, GOT, SCS		[73, 76, 88, 118]	
*Pseudaxine vietnamensis* (Lebedev, Parukhin & Roitman, 1970) Bouguerche, Tazerouti, Gey & Justine, 2020		Mo			*Caranx* sp., *Decapterus* sp., *Selar crumenophthalmus*, *Selaroides leptolepis*, *Seriola dumerili*		Gi	QN, GOT, SCS		[73, 76, 88, 118]	
*Sibitrema poonui* Yamaguti, 1966		Mo			*Auxis thazard*, *Euthynnus affinis*, *T. thynnus*		Gi	SCS		[74, 80]	
**Family: Gotocotylidae Yamaguti, 1963**											
*Cathucotyle cathuaui* Lebedev, 1968		Mo			*Scomberomorus commerson*, *S. guttatus.*		Gi	GOT, SCS		[72, 73]	
*Gotocotyla acanthura* (Parona & Perugia, 1896) Meserve, 1938		Mo			*Scomberomorus guttatus*		Gi	GOT		[73]	
*Gotocotyla bivaginalis* (Ramalingam, 1961) Rohde, 1976		Mo			*Scomberomorus commerson*		Gi	GOT		[74]	
*Gotocotyla laticauda* Lebedev, 1970		Mo			*Scomberomorus commerson*		Gi	GOT		[73]	
**Family: Heteraxinidae Unnithan, 1957**											
*Bicotyle perpolita* Lebedev, 1968		Mo			*Pampus argenteus*, *Parastromateus niger*		Gi	GOT, SCS		[69, 81]	
*Heteraxine heterocerca* (Goto, 1894) Yamaguti, 1938		Mo			*Caranx* sp., *Selar crumenophthalmus*		Gi, Phc	GOT, SCS		[69, 81]	
*Kannaphallus virilis* Unnithan, 1957		Mo			*Alectis indicus*, *Carangoides malabaricus*,		Gi	SCS, GOT		[73, 76, 118]	
*Karvolicola ruber* Nguyen, Nguyen, Tatonova, 2020		Mo			*Otolithes ruber*		Gi	QB		[94]	
*Karvolicola tuyeti* Nguyen, Nguyen, Tatonova, 2020		Mo			*Nibea albiflora*		Gi	QB		[94]	
*Lethrinaxine parva* Mamaev, 1970		Mo			*Gymnocranius griseus*		Gi	GOT		[81]	
*Monaxine formionis* Unnithan, 1957		Mo			*Parastromateus niger*		Gi	GOT		[81]	
**Family: Heteromicrocotylidae Unnithan, 1961**											
*Heterapta chorinemi* (Tripathi, 1956) Unnithan, 1961		Mo			*Acanthopagrus pacificus*		Gi	QN		[93]	
*Heteromicrocotyla carangis* Yamaguti, 1953		Mo			*Carangoides malabaricus*, *Seriola* sp., *Seriolina nigrofasciata,* Carangidae gen. sp.		Gi	GOT, SCS		[69, 73, 76, 118]	
*Heteromicrocotyla polyorchis* Unnithan, 1961		Mo			Carangidae gen. sp.		Gi	GOT, SCS		[73, 76, 118]	
*Heteromicrocotyla vaginispina* Unnithan, 1961		Mo			*Carangoides malabaricus*		Gi	GOT, SCS		[73, 118]	
*Heteromicrocotyla* sp.		Mo			Carangidae gen. sp., *Carangoides malabaricus*, *Decapterus* sp., *Selaroides leptolepis*		Gi	GOT, SCS		[73, 76, 118]	
**Family: Hexostomatidae Price, 1936**											
*Hexostoma thynni* (Delaroche, 1811) Rafinesque, 1815		Mo			*Auxis thazard*		Gi	SCS		[80]	
*Neohexostoma euthynni* (Meserve, 1938) Price, 1961		Mo			*Auxis thazard*, *Euthynnus affinis*		Gi	SCS		[80]	
**Family: Mazocraeidae Price, 1936**											
*Neomazocraes dorosomatis* (Yamaguti, 1938) Price, 1943		Mo			*Clupanodon thrissa*		Gi	QN		[88]	
*Paramazocraes thrissocles* Tripathi, 1959		Mo			*Thrissocles* sp.		Gi	GOT			
**Family: Microcotylidae Taschenberg, 1879**											
*Caballeraxine chainanica* (Lebedev, Parukhin & Roitman, 1970) Lebedev, 1972		Mo			*Carangoides malabaricus*		Gi	GOT, SCS		[118]	
*Diplostamenides sciaenae* (Goto, 1894) Mamaev, 1986		Mo			*Seriola* sp.		Gi	GOT, SCS		[73, 118]	
*Incisaxine dubia* Mamaev, 1970		Mo			*Gerres* sp.		Gi	GOT		[81]	
*Intracotyle orientalis* Mamaev, 1970		Mo			*Pomadasys argenteus*		Gi	GOT		[81]	
*Lutianicola haifonensis* Lebedev, 1970		Mo			*Lutjanus russellii*, *L. sebae*		Gi	GOT		[73]	
*Microcotyle* sp.		Mo			*Carangoides malabaricus*		Gi	GOT		[73]	
*Polylabroides* cf. *guangdongensis* Zhang & Yang, 2000		Mo			*Acanthopagrus latus*		Gi	QN		Present study	
*Polylabroides tienyenensis* Nguyen, Nguyen, Ha, Ngoc, Ngoc, Le, Tatonova & Greiman, 2020		Mo			*Acanthopagrus pacificus*		Gi	QN		[93]	
*Polylabroides tonkinensis* Nguyen, Nguyen, Ha, Ngoc, Ngoc, Le, Tatonova & Greiman, 2020		Mo			*Acanthopagrus pacificus*		Gi	QN		[93]	
*Tonkinaxine homocerca* Lebedev, Parukhin & Roitman, 1970		Mo			*Carangoides malabaricus*, *Seriola dumerili*, *Seriola* sp., *Seriolina nigrofasciata*		Gi	GOT, SCS		[76, 118]	
**Family: Plectanocotylidae Monticelli, 1903**											
*Triglicola tonkinensis* Mamaev & Parukhin, 1972		Mo			*Lepidotrigla* sp.		Gi	GOT		[84, 119]	
**Family: Protomicrocotylidae Johnston & Tiegs, 1922**											
*Bilaterocotyloides carangis* Ramalingam, 1961		Mo			Carangidae gen. sp., *Megalaspis cordyla*		Gi	GOT, SCS		[73, 118]	
*Bilaterocotyloides madrasensis* Radha, 1966		Mo			*Megalaspis cordyla*		Gi	GOT		[73, 76, 118]	
*Vallisiopsis contorta* Subhapradha, 1951		Mo			*Lactarius lactarius*		Gi	GOT, SCS		[74]	
**Family: Thoracocotylidae Price, 1936**											
*Pricea multae* Chauhan, 1945		Mo			*Scomberomorus commerson*, *S. guttatus*		Gi	GOT		[73]	
*Pseudothoracocotyla ovalis* (Tripathi, 1956) Yamaguti, 1963		Mo			*Scomberomorus commerson*, *S. guttatus*		Gi	GOT, SCS		[73, 119]	
Monogenea gen. sp.		Mo			*Abalistes stellaris*		Gi	SCS		[119]	
**Class: Trematoda Rudolphi, 1808**											
**Subclass: Digenea Carus, 1863**											
**Order: Diplostomida Olson, Cribb, Tkach, Bray & Littlewood, 2003**											
**Suborder: Diplostomata Olson, Cribb, Tkach, Bray & Littlewood, 2003**											
**Superfamily: Schistosomatoidea Stiles & Hassall, 1898**											
**Family: Aporocotylidae Odhner, 1912**											
*Cardicola congruentus* Lebedev & Mamaev, 1968		D			*Euthynnus affinis*		Bvg	GOT		[75]	
*Cardicola grandis* Lebedev and Mamaev, 1968		D			*Makaira* sp.		Iw	GOT		[75]	
*Cardallagium* cf. *anthicum* (Bullard & Overstreet, 2006) Yong, Cutmore, Jones, Gauthier & Cribb, 2017		D			*Rachycentron canadum*		He	KH		[141]	
**Order: Plagiorchiida La Rue, 1957**											
**Suborder: Apocreadiata Olson, Cribb, Tkach, Bray & Littlewood, 2003**											
**Superfamily: Apocreadioidea Skrjabin, 1942**											
**Family: Apocreadiidae Skrjabin, 1942**											
*Homalometron* sp.		D			*Gerres filamentosus*		In	GOT		[81]	
*Schistorchis skrjabini* Parukhin, 1963		D			*Abalistes stellaris*, *Triacanthus biaculeatus*		In	GOT		[106, 119]	
*Sphincteristomum acollum* Oshmarin et al., 1961		D			*Abalistes stellaris*		In	GOT		[99, 119]	
**Suborder: Bivesiculata Olson, Cribb, Tkach, Bray & Littlewood, 2003**											
**Superfamily: Bivesiculoidea Yamaguti, 1934**											
**Family: Bivesiculidae Yamaguti, 1934**											
*Bivesicula claviformis* Yamaguti, 1934		D			*Amphiprion clarckii*, *A. perideraion; A. polymmus*		S	KH		[27]	
*Bivesicula* sp., Metacercaria		D			*Amphiprion clarckii*, *A. perideraion*		In	KH		[142]	
*Paucivitellosus vietnamensis* Atopkin, Besprozvannykh, Ngo, Van Ha, Van Tang, Ermolenko & Beloded, 2016		D			*Liza subviridis*		In	GOT		[20]	
**Suborder: Bucephalata La Rue, 1926**											
**Superfamily: Bucephaloidea Poche, 1907**											
**Family: Bucephalidae Poche, 1907**											
*Alcicornis baylisi* Nagaty, 1937		D			*Carangoides malabaricus*		In	SCS		[113]	
*Alcicornis carangis* MacCallum, 1917		D			*Caranx* sp.		In	GOT, SCS		[71, 73]	
*Bucephalus fragilis* Velasquez, 1959		D			*Megalaspis cordyla*, *Scomberoides lysan*		In	SCS		[113]	
*Bucephalus gorgon* (Linton, 1905) Eckmann, 1932		D			*Seriolina nigrofasciata*		S	GOT		[95]	
*Bucephalus introversus* Manter, 1940		D			*Seriolina nigrofasciata*		In	SCS		[118]	
*Bucephalus varicus* Manter, 1940		D			*Atropus atropos*, *Atule mate*, *Rachycentron canadum*, *Selar crumenophthalmus*		S, In	SCS, GOT		[113, 117, 118]	
*Bucephalus paraheterotentaculatus* Velasquez, 1959		D			*Seriola dumerili*, *Seriolina nigrofasciata*		In	SCS		[113, 118]	
*Bucephalus polymorphus* von Baer, 1827		D			*Lates calcarifer*		In	KH		[140]	
*Bucephalus* sp.		D			*Gerres filamentosus*		S	SCS, GOT		[81]	
*Prosorhynchus epinepheli* Yamaguti, 1939		D			*Epinephelus coioides*, *E. bleekeri*, *E. bruneus*, *E. malabaricus*, *E. sexfasciatus*, *E. tauvina, Lutjanus argentimaculatus*		In	KH, QN		[34, 90, 140]	
*Prosorhynchus pacificus* Manter, 1940		D			*Epinephelus bleekeri*, *E. coioides*, *E. malabaricus*		In	KH		[135]	
*Prosorhynchus luzonicus* Velasquez, 1959		D			*Epinephelus coioides*		S, In, Py	HP, ND, QN, TH		[132]	
*Prosorhynchus tonkinensis* Truong, Palm, Bui, Thuy Ngo & Bray, 2016		D			*Epinephelus coioides*		S, Py	ND		[132]	
*Prosorhynchus maternus* Bray & Justine, 2006		D			*Epinephelus coioides*		S	HP		[132]	
*Prosorhynchus* sp. A^2^		D			*Epinephelus coioides*		S	HP		[132]	
*Prosorhynchus* sp. B^3^		D			*Epinephelus coioides*		S	HP		[132]	
*Prosorhynchus* sp.		D			*Acanthopagrus latus*, *Auxis thazard*		In	NA, QB, QN SCS		[80, 90]; Present study	
*Rhipidocotyle laruei* Velasquez, 1959		D			*Psettodes erumei*		S, In	SCS, GOT		[114, 119]	
*Rhipidocotyle pentagonum* (Ozaki, 1924) Eckmann, 1932		D			*Auxis thazard*, *Euthynnus affinis*, *Thunnus thynnus*		S, In	SCS		[95]	
*Rhipidocotyle* sp.		D			*Drepane punctata*, *Ephippus orbis*, *Gerres filamentosus*, *Gymnocranius griseus*, *Leiognathus equulus*, *Parastromateus niger*, Leiognathidae gen. sp., Sciaenidae gen. sp.		Gi, Go, In, K, Ve	GOT, SCS		[81, 95, 133]	
*Prosorhynchinae* gen. sp.		D			*Epinephelus coioides*		In	QN		[133]	
**Superfamily: Gorgoderoidea Looss, 1901**											
**Family: Callodistomidae Odhner, 1910**											
*Cholepotes* sp.		D			*Parupeneus multifasciatus*		In	KH		[27]	
**Superfamily: Gymnophalloidea Odhner, 1905**											
**Family: Fellodistomidae Nicoll, 1909**											
*Complexobursa vjetnamensis* Oshmarin & Mamaev, 1963		D			*Terapon theraps*		In	GOT		[98]	
*Lintonium vibex* (Linton, 1900) Stunkard & Nigrelli, 1930		D			*Abalistes stellaris*, *Aluterus monoceros*, *Monacanthus chinensis*, *Scomberoides lysan*, *Scomberomorus commerson*		In, L	GOT, SCS		[73, 95, 113]	
*Proctoeces* sp*.*		D			*Acanthopagrus latus*		In	QN		Present study	
*Pseudosteringophorus* sp.		D			*Ephippus orbis*		In	GOT		[81]	
*Tergestia laticollis* (Rudolphi, 1819) Stossich, 1899		D			*Alepes melanoptera*, *Decapterus* sp., *Megalaspis cordyla*, *Selar crumenophthalmus*, *Selaroides leptolepis*		In	SCS		[118]	
**Family: Tandanicolidae Johnston, 1927**											
*Buckleytrema indicum* Gupta, 1956		D			*Arius* sp.		In	GOT		[98]	
*Monodhelmis torpedinis* Dollfus, 1937		D			*Arius arius*		In	HP		[90]	
**Suborder: Haploporata Pérez-Ponce de León & Hernández-Mena, 2019**											
**Superfamily: Haploporoidea Nicoll, 1914**											
**Family: Haploporidae Nicoll, 1914**											
*Parahaploporus elegantus* Atopkin, Besprozvannykh, Ha, Nguyen & Nguyen, 2019		D			*Osteomugil cunnesius*		In	KH		[23]	
*Parasaccocoelium mugili* Zhukov, 1971		D			*Liza haematocheila*		In	QN, HP		[23]	
*Pseudohaploporus vietnamensis* Atopkin, Besprozvannykh, Ha, Tang, Nguyen, Nguyen & Chalenko, 2018		D			*Moolgarda seheli*, *Osteomugil engeli*		In	GOT		[22]	
*Pseudohaploporus planilizum* Atopkin, Besprozvannykh, Ha, Nguyen, Nguyen & Chalenko, 2018		D			*Planiliza subviridi*		In	GOT		[22]	
*Pseudohaploporus pusitestis* Atopkin, Besprozvannykh, Ha, Nguyen & Nguyen, 2019		D			*Moolgarda seheli*			HP		[23]	
*Pseudohaploporus* sp.		D			*Moolgarda seheli*		In	GOT		[22]	
*Skrjabinolecithum spasskii* Belous, 1954		D			*Liza haematocheila*, *Mugil cephalus*		In	HP		[90]	
**Suborder: Haplosplanchnata Olson, Cribb, Tkach, Bray & Littlewood, 2003**											
**Superfamily: Haplosplanchnoidea Poche, 1925**											
**Family: Haplosplanchnidae Poche, 1926**											
*Haplosplanchnus pachysoma* (Eysenhardt, 1829) Looss, 1902		D			*Liza engeli*		In	QN		[24]	
**Suborder: Hemiurata Skrjabin & Guschanskaja, 1954**											
**Superfamily: Hemiuroidea Looss, 1899**											
**Family: Accacoeliidae Odhner, 1911**											
*Tetrochetus hansoni* (Parukhin, 1964) Hafeezullah, 1982		D			*Aluterus monoceros*		In	GOT		[107]	
*Tetrochetus* sp.		D			*Nemipterus hexodon*		S	KH		[27]	
**Family: Bathycotylidae**											
*Bathycotyle* sp.		D			*Pampus argenteus*		Gic	GOT		[73]	
**Family: Derogenidae Nicoll, 1910**											
*Derogenes varicus* (Müller, 1784) Looss, 1901		D			*Rachycentron canadum*, *Triacanthus biaculeatus*		S, In	GOT		[117, 119]	
*Gonocercella pacifica* Manter, 1940		D			*Drepane punctata*		In	GOT		[81]	
*Gonocercella* sp.		D			*Atropus atropos*, *Psettodes erumei*, *Scomberoides lysan*		In	GOT, SCS		[113, 118]	
**Family: Dictysarcidae Skrjabin & Guschanskaja, 1955**											
*Elongoparorchis pneumatis* Rao, 1961		D			*Arius* sp.		Sb	SCS		[96]	
**Family: Didymozoidae Monticelli, 1888**											
*Colocyntotrema auxis* Yamaguti, 1951		D			Scombridae gen. sp.		Bc, In	SCS		[80]	
*Didymodiclinus epinepheli* (Abdul-Salam, Sreelatha & Farah, 1990) Pozdnyakov, 1994		D			*Epinephelus coioides*		Mu	KH		[135]	
*Lobatozoum multisacculatum* Ishii, 1935		D			*Auxis thazard*		Iw, S, Ve	SCS		[80]	
*Metanematobothrium bivitellatum* Mamaev, 1968		D			*Auxis thazard*, *Euthynnus affinis*		Bc, Bvg, L, K	SCS		[80]	
*Monilicaecum ventricosum* Yamaguti, 1942		D			*Abalistes stellaris*, *Psettodes erumei*		Bvg, L	GOT		[119]	
*Multtubovarium amphibolum* Mamaev, 1970		D			*Platax orbicularis*		Gi, Ht	GOT		[81]	
*Nematobothrium* sp.		D			*Euthynnus affinis*, *Thunnus thynnus*		L, Phc	SCS		[80]	
*Neometadidymozoon polymorphis* (Oschmarin & Mamaev, 1963) Yamaguti, 1971		D			*Priacanthus tayenus*		Bc, Gi, Mu	GOT		[98]	
*Neometanematobothrioides rachycentri* (Parukhin, 1969) Yamaguti, 1971		D			*Rachycentron canadum*		Bc, Gi	GOT, SCS		[116, 118]	
*Oesophagocystis dissimilis* (Yamaguti, 1938) Yamaguti, 1970		D			*Auxis thazard*, *Euthynnus affinis*, *Thunnus thynnus*		O, S, In	SCS		[80]	
*Torticaecum fenestratum* (Linton, 1907) Yamaguti, 1942		D			*Psettodes erumei*, *Triacanthus biaculeatus*		Bvg, L	GOT		[119]	
Didymozoidae gen. sp.		D			*Atule mate*, *Auxis thazard*, *Carangoides malabaricus*, *Echeneis naucrates*, *Epinephelus coioides*, *Euthynnus affinis*, *Leiognathus equulus*, *Psettodes erumei*, *Rachycentron canadum*, *Selar crumenophthalmus*, *Seriolina nigrofasciata*, *Thunnus thynnus,* Leiognathidae gen. sp.		Bc, Ve, Gi, In, K, L	GOT, SCS		[80, 81, 113, 115, 117, 133]	
**Family: Hemiuridae Looss, 1899**											
*Allostomachicola secundus* (Srivastava, 1939) Yamaguti, 1958		D			*Chirocentrus dorab*		S	GOT		[81]	
*Aphanurus mugilus* Tang 1981		D			*Moolgarda engeli*		I	HP		[21]	
*Aphanurus stossichii* (Monticelli, 1891) Looss, 1907		D			*Drepane punctata*, *Ephippus orbis*, *Pampus argenteus*, *Thunnus thynnus*		S, In	GOT, SCS		[73, 80, 81]	
*Aphanurus* sp.		D			*Mugil* sp.		In	GOT		[90]	
*Dinurus euthynni* Yamaguti, 1934		D			*Auxis thazard*, *Euthynnus affinis*, *Thunnus* sp.			SCS		[80]	
*Dinurus longisinus* Looss, 1907		D			*Carangoides malabaricus*		S	GOT		[113, 118]	
*Dinurus selari* Parukhin, 1966		D			*Atropus atropos*, *Atule mate*, *Carangoides malabaricus*, *Decapterus* sp., *Rachycentron canadum*, *Selar crumenophthalmus*, *Selaroides leptolepis,* Carangidae gen. sp.		S, In	GOT, SCS		[19, 111, 113, 118]	
*Dinurus* sp.		D			*Mene maculata*		S	GOT		[81]	
*Ectenurus selari* (Parukhin, 1966) Yamaguti, 1971		D			*Atule mate*, *Caranx* sp., *Epinephelus bruneus*, *E. coioides*, *E. sexfasciatu*s, E. tauvina*, *Megalaspis cordyla*, *Rachycentron canadum*, *Selar crumenophthalmus*, *Selaroides leptolepis,* Carangidae gen. sp.		S, In	GOT, SCS		[34, 73, 112, 113, 118, 121]	
*Ectenurus theraponae* Oshmarin, 1965		D			*Terapon theraps*		S	GOT		[95]	
*Ectenurus trachuri* (Yamaguti, 1934) Yamaguti, 1970		D			*Caranx* sp., *Selar crumenophthalmus,* Carangidae gen. sp.		S	GOT, SCS		[71, 73]	
*Ectenurus* sp.		D			*Selar crumenophthalmus*		S	GOT		[95]	
*Erilepturus formosae* Reid, Coil & Kuntz, 1966		D			*Decapterus* sp*.*, *Dussumieria elopsoides*		S	GOT		[73, 81]	
*Erilepturus hamati* (Yamaguti, 1934) Manter, 1947		D			*Eleutheronema tetradactylum*, *Epinephelus bleekeri*, *E. coioides*, *E. malabaricus*, *Platycephalus indicus*, *Nibea albiflora*,		In	NA		[90, 138]	
*Erilepturus neopacificus* (Velasquez, 1962) Gupta & Jain, 1992		D			*Lates calcarifer*		Gi	KH		[122]	
*Erilepturus* sp.		D			*Acanthopagrus latus*, Sciaenidae gen. sp.		S	GOT, HL		[95]; Present study	
*Elytrophalloides* sp.		D			*Thryssa mystax*		S	ND		[90]	
*Hemiurus arelisci* Yamaguti, 1938		D			*Scomberoides lysan*		S	QN, ND		[88, 90]	
*Lecithochirium alectis* Yamaguti, 1970		D			*Nibea albiflora*		In	QN, ND		[88, 90]	
*Lecithochirium imocavum* (Looss, 1907) Skrjabin & Guschanskaja, 1955		D			*Auxis thazard*, *Euthynnus affinis*, *Ilisha* sp., *Thunnus thynnus*, *Thunnus* sp.		S	GOT		[80, 81]	
*Lecithochirium magnaporum* Manter, 1940		D			*Atropus atropos*		S	SCS		[113]	
*Lecithochirium microstomum* Chandler, 1935		D			*Atropus atropos*		S	SCS		[113]	
*Lecithochirium monticellii* (Linton, 1898) Skrjabin & Guschanskaja, 1955		D			*Amphiprion clarkia*, *Atropus atropos*, *Echeneis naucrates*, *Selar crumenophthalmus*		S	KH, SCS		[27, 113, 115]	
*Lecithochirium* sp.		D			*Mene maculata*, *Seriolina nigrofasciata*		S	GOT, SCS		[81, 113]	
*Lecithocladium apolecti* Velasquez, 1962		D			*Ephippus orbis*, *Gerres filamentosus*, *Parastromateus niger*, *Rastrelliger kanagurta,* Leiognathidae gen. sp.		S, In	GOT		[73, 81]	
*Lecithocladium excisiforme* Cohn, 1902		D			*Alepes melanoptera*, *Caranx* sp., *Selaroides leptolepis*		S	GOT, SCS		[118]	
*Lecithocladium excisum* (Rudolphi, 1819) Lühe, 1901		D			*Alepes melanoptera*, *Caranx* sp., *Decapterus maruadsi*, *Decapterus* sp., *Elentheronema tetradactylum*, *Sardinella* sp., *Selar crumenophthalmus*, *Scomberoides lysan*		S	GOT, SCS		[73, 81, 88, 118]	
*Lecithocladium harpodontis* Srivastava, 1942		D			*Atropus atropos*, *Decapterus* sp., *Dussumieria elopsoides*, *Ilisha* sp., *Selar crumenophthalmus*		S	GOT, SCS		[81, 113, 118]	
*Lecithocladium megalaspis* Yamaguti, 1953		D			*Megalaspis cordyla*		S, In	SCS		[113]	
*Lecithocladium pampi* Lebedev, 1968		D			*Pampus argenteus*		In	GOT, SCS		[70, 73]	
*Lecithocladium seriolellae* Manter, 1954		D			*Carangoides malabaricus*, *Selar crumenophthalmus*		S, In	SCS		[113, 118]	
*Lecithocladium* sp.		D			*Mene maculata*		S	GOT		[81]	
*Merlucciotrema praeclarum* (Manter, 1934) Yamaguti, 1971		D			*Platycephalus indicus*, *Eleutheronema tetradactylum*		S, In	QN, ND		[90]	
*Parahemiurus clupeae* Yamaguti, 1953		D			*Herklotsichthys quadrimaculatus*		S	KH		[64]	
*Parahemiurus merus* (Linton, 1910) Manter, 1940		D			*Atropus atropos*, *Decapterus* sp., *Sardinella* sp., *Scomberoides lysan*		S, In	GOT, SCS		[81, 118]	
*Parahemiurus* sp.		D			*Alepes kleinii*		S	QN		[88]	
*Plerurus digitatus* (Looss, 1899) Looss, 1907		D			*Auxis thazard*, *Euthynnus affinis*, *Thunnus* sp., Carangidae gen. sp.			GOT, SCS		[80, 113, 118]	
*Stomachicola muraenesocis* Yamaguti, 1934		D			*Congresox talabonoides*		S	QN (QB)		[88, 90]	
*Tubulovesicula angusticauda* (Nicoll, 1915) Yamaguti, 1934		D			*Epinephelus merra*, *Rachycentron canadum*, *Trachinocephalus* sp.		S, In	GOT, SCS		[64, 95, 118]	
*Tubulovesicula lindbergi* (Layman, 1930) Yamaguti, 1934		D			*Echeneis naucrates*, *Psettodes erumei,* Carangidae gen. sp.		S	GOT, SCS		[115, 118, 119]	
*Tubulovesicula marsupialia* Oshmarin, 1965		D			*Saurida tumbil*		In	GOT		[95]	
*Tubulovesicula* sp.		D			*Trachinocephalus myops*		S, In	KH		[27]	
Hemiuridae gen. sp.		D			*Euthynnus affinis*, *Thunnus thynnus*, *Thunnus* sp.			SCS		[80]	
**Family: Hirudinellidae Dollfus, 1932**											
*Hirudinella ventricosa* (Pallas, 1774) Baird, 1853		D			*Euthynnus affinis*, *Seriolina nigrofasciata*, *Thunnus thynnus*		S	SCS		[80, 113]	
**Family: Lecithasteridae Odhner, 1905**											
*Aponurus carangis* Yamaguti, 1952		D			*Decapterus* sp., *Rachycentron canadum*, *Selar crumenophthalmus*		S, In	GOT, SCS		[73, 113, 118]	
*Aponurus laguncula* Looss, 1907		D			*Amphiprion polymnus*, *Atropus atropos*, *Drepane punctata*, *Dussumieria elopsoides*, *Ilisha* sp., *Leiognathus* sp., *Megalaspis cordyla*, *Parastromateus niger*, *Sardinella* sp., *Selar crumenophthalmus*, *Seriolina nigrofasciata*, *Thunnus* sp., Carangidae gen. sp.		In, S	GOT, SCS		[80, 81, 113, 118, 142]	
*Aponurus pyriformis* (Linton, 1910) Overstreet, 1973		D			*Amphiprion clarckii*, *Parastromateus niger*, *Platax orbicularis*		In, S	GOT, KH		[81, 142]	
*Aponurus* sp.		D			*Epinephelus coioides*		S	GOT		[133]	
*Hysterolecitha nahaensis* Yamaguti, 1942		D			*Amphiprion clarckii*, *A. frenatus*, *A. perideraion*, *A. polymnus*, *Dascyllus trimaculatus*		S	SCS		[64, 142]	
*Lecithaster mugilis* Yamaguti, 1970		D			*Moolgarda engeli*, *M. seheli*, *Liza subviridis*		In	GOT		[25]	
*Lecithaster stellatus* Looss, 1907		D			*Seriolina nigrofasciata*		S	SCS		[113]	
*Trifoliovarium triacanthi* (Parukhin, 1964) Bray & Cribb, 2000		D			*Triacanthus biaculeatus*		In	GOT		[108]	
**Family: Sclerodistomidae Odhner, 1927**											
*Prosogonotrema bilabiatum* Vigueras, 1940		D			*Abalistes stellaris*, *Ephippus orbis*, *Platax orbicularis*		S	GOT		[81, 107, 119]	
*Prosogonotrema symmetricum* Oshmarin, 1965		D			*Lutjanus* sp., *Pristipomoides typus*		S	GOT		[95, 118]	
*Prosorchis chainanensis* Lebedev, 1970		D			*Ephippus orbis*, *Pampus argenteus*, *Parastromateus niger*		S	GOT		[73, 81]	
Hemiuroidea gen. sp.		D			*Alectis indicus*, *Gnathanodon speciosus*, *Megalaspis cordyla*, *Rachycentron canadum*, *Seriolina nigrofasciata*, *Terapon theraps,* Carangidae gen. sp.		In	GOT, SCS		[95, 113, 117]	
**Suborder: Lepocreadiata Olson, Cribb, Tkach, Bray & Littlewood, 2003**											
**Superfamily: Lepocreadioidea Odhner, 1905**											
**Family: Aephnidiogenidae Yamaguti, 1934**											
*Aephnidiogenes barbarus* Nicoll, 1915		D			*Pomadasys argenteus*		In	GOT		[81]	
**Family: Gyliauchenidae Fukui, 1929**											
*Gyliauchen tarachodes* Nicoll, 1915		D			*Siganus fuscescens*		In	HP		[90]	
**Family: Lepocreadiidae Odhner, 1905**											
*Diploproctia drepanei* Mamaev, 1970		D			*Drepane punctata*		In	GOT		[81, 90]	
*Diploproctodaeum haustrum* (MacCallum, 1919) La Rue, 1926		D			*Aluterus monoceros*		In	QB		[90]	
*Diploproctodaeoides longipygum* (Oshmarin, Mamaev & Parukhin, 1961) Reimer, 1981		D			*Abalistes stellaris*		In	GOTh, GOT		[99]	
*Diploproctodaeum macracetabulum* Oshmarin, Mamaev & Parukhin, 1961		D			*Abalistes stellaris*, *Triacanthus biaculeatus*		In	GOTh, GOT		[99]	
*Diploproctodaeum plataxi* Mamaev, 1970		D			*Platax orbicularis*		In	GOT		[81]	
*Diploproctodaeum rutellum* (Mamaev, 1970) Bray, Cribb & Barker, 1996		D			*Ephippus orbis*, *Platax orbicularis*		In	GOT		[81]	
*Echeneidocoelium indicum* Simha & Pershad, 1964		D			*Echeneis naucrates*, *Psettodes erumei*		In, S	GOT, SCS		[112, 119]	
*Hypocreadium cavum* Bray & Cribb, 1966		D			*Abalistes stellaris*		In	GOT		[95]	
*Hypocreadium scaphosomum* (Manter, 1940) Bravo Hollis & Manter, 1957		D			*Abalistes stellaris*, *Aluterus monoceros*, *Triacanthus biaculeatus*		In	GOTh, GOT		[119]	
*Hypocreadium* sp.		D			*Abalistes stellaris*			GOTh		[119]	
*Lepidapedon megalaspi* Parukhin, 1966		D			*Carangoides malabaricus*, *Decapterus* sp., *Megalaspis cordyla*, *Rachycentron canadum*		In	GOT, SCS		[112, 113]	
*Lepocreadium* sp.		D			*Ephippus orbis*, *Triacanthus biaculeatus*		In	GOT		[81, 119]	
*Multitestis magnacetabulum* Mamaev, 1970		D			*Ephippus orbis*, *Platax orbicularis*		In	GOT		[81]	
*Neoallolepidapedon fistulariae* (Oshmarin, 1965) Yamaguti, 1965		D			*Fistularia petimba*		S	GOT		[96]	
*Opechona formiae* Oshmarin, 1965		D			*Pampus argenteus*, *Parastromateus niger,* Leiognathidae gen. sp.		S, In	GOT		[73, 95]	
*Trigonotrema alatum* Goto & Ozaki, 1929		D			*Brachiostegus japonicus*, *Drepane longimana*, *D. punctata*, *Ephippus orbis*		In, S	GOT		[95]	
Lepocreadiidae gen. sp.		D			*Plectorhinchus cinctus*		In	GOT		[81]	
**Suborder: Monorchiata Olson, Cribb, Tkach, Bray & Littlewood, 2003**											
**Superfamily: Monorchioidea Odhner, 1911**											
**Family: Monorchiidae Odhner, 1911**											
*Alloinfundiburictus cacuminatus* (Nicoll, 1915) Wee, Cutmore, Pérez-del-Olmo & Cribb, 2020		D			*Pomadasys argenteus*		In, S	GOT		[95]	
*Alloinfundiburictus cryptostoma* (Oshmarin, 1966) Wee, Cutmore, Pérez-del-Olmo & Cribb, 2020		D			*Pomadasys argenteus*		In, S	GOT		[81]	
*Ancylocoelium tropicum* (Manter, 1940) Wee, Cutmore, Pérez-del-Olmo & Cribb, 2020		D			*Carangoides malabaricus*, *Megalaspis cordyla*		In	GOT		[81]	
*Hurleytrematoides chaetodoni* (Manter, 1942) Yamaguti, 1954		D			*Chaetodon* sp.		In	GOT		[81]	
*Huridostomum formionis* Mamaev, 1970		D			*Parastromateus niger*		In	GOT		[81]	
*Infundiburictus chaetodipteri* (Thomas, 1959) Wee, Cutmore, Pérez-del-Olmo & Cribb, 2020		D			*Pomadasys argenteus*		In, S	GOT		[81]	
*Leiomonorchis mamaevi* Madhavi, 2008		D			*Leiognathus equulus*, *Parastromateus niger,* Leiognathidae gen. sp.		In	GOT		[81]	
*Monorcheides diplorchis* Odhner, 1905		D			*Scolopsis taeniopterus*		I	KH		[27]	
*Monorchis diplovarium* Mamaev, 1970		D			*Pomadasys argenteus*		In	GOT		[81]	
*Opisthomonorcheides decapteri* Parukhin, 1966		D			*Atule mate*, *Decapterus* sp.		In	GOT, SCS		[112, 113]	
*Opisthomonorcheides ovacutus* (Mamaev, 1970) Machida, 2011		D			*Parastromateus niger*		In	GOT		[81]	
*Opisthomonorchis carangis* Yamaguti, 1952		D			*Carangoides malabaricus,* Carangidae gen. sp.		In	GOT, SCS		[118]	
*Paralasiotocus macrorchis* (Yamaguti, 1934) Wee, Cutmore, Pérez-del-Olmo & Cribb, 2020		D			*Plectorhinchus cinctus*, *Plectorhinchus* sp.		In	GOT		[81]	
*Proctotrema* sp.		D			*Pomadasys argenteus*, *Selar crumenophthalmus*		In	GOT, SCS		[95, 113, 119]	
*Sinistroporomonorchis lizae* (Liu, 2002) Wee, Cutmore, Pérez-del-Olmo & Cribb, 2020		D			*Moolgarda cunnesius, Liza engeli, L. longimanus, L. subviridis, Valamugil seheli*		In	GOT		[20]	
**Suborder: Opisthorchiata La Rue, 1957**											
**Superfamily: Opisthorchioidea Looss, 1899**											
**Family: Cryptogonimidae Ward, 1917**											
*Beluesca plectorhyncha* (Mamaev, 1970) Miller & Cribb, 2007		D			*Plectorhinchus cinctus*		In	GOT		[81]	
*Metadena eurystoma* Oshmarin, 1965		D			Sciaenidae gen. sp.		In	GOT		[95]	
*Metadena longa* (Oshmarin, Mamaev & Parukhin, 1961) Miller & Cribb, 2008		D			*Pristipomoides typus*		In	GOT		[99]	
*Metadena* sp.		D			*Lutjanus russellii*		In	QB		[90]	
*Neometadena ovata* (Yamaguti, 1952) Miller & Cribb, 2008		D			*Lutjanus russellii*		In	QB		[90]	
*Pseudometadena celebesensis* Yamaguti, 1952		D			*Lates calcarifer*		In	KH		[140]	
*Siphoderina echinostomus* (Oshmarin, Mamaev & Parukhin, 1961) Miller & Cribb, 2008		D			*Pristipomoides typus*		In	GOT		[99]	
*Siphoderina morosovi* (Parukhin, 1965) Miller & Cribb, 2008		D			*Rachycentron canadum*		In	GOT, SCS		[110, 118]	
*Siphoderina* sp.		D			*Lutjanus argentimaculatus*		In	QB		[90]	
**Family: Heterophyidae Leiper, 1909**											
*Centrocestus* sp.		D			*Epinephelus coioides*		Mu, Fi	GOT		[133]	
*Heterophyopsis continua* (Onji & Nishio, 1916) Price, 1940		D			*Epinephelus bleekeri*, *E. coioides*		Mu, Fi	KH		[130, 135, 137]	
*Procerovum varium* Onji & Nishio, 1916		D			*Epinephelus bleekeri*, *E. coioides*		Mu, Fi	KH		[135]	
Heterophyidae gen. sp.		D			*Epinephelus coioides*, *Protonibea dicanthus*, *Sardinella* sp.		In	GOT, SCS		[81, 133]; Present study	
**Suborder: Pronocephalata Skrjabin, 1955**											
**Superfamily: Paramphistomoidea Fischoeder, 1901**											
**Family: Cladorchiidae Fischoeder, 1901**											
*Cleptodiscus* sp.		D			*Triacanthus biaculeatus*		In	GOT		[119]	
**Suborder: Transversotremata Olson, Cribb, Tkach, Bray & Littlewood, 2003**											
**Superfamily: Transversotrematoidea Witenberg, 1944**											
**Family: Transversotrematidae Witenberg, 1944**											
*Transversotrema patialense* (Soparkar, 1924) Crusz & Sathananthan, 1960		D			*Epinephelus bleekeri*, *E. coioides*		Us	KH, ND		[133, 140]	
**Suborder: Xiphidiata Olson, Cribb, Tkach, Bray & Littlewood, 2003**											
**Superfamily: Brachycladioidea Odhner, 1905**											
**Family: Acanthocolpidae Lühe, 1906**											
*Acanthocolpus liodorus* Lühe, 1906		D			*Chirocentrus dorab*, *Ilisha* sp., *Sardinella* sp.		Ve	GOT		[82]	
*Pleorchis hainanensis* Shen, 1983		D			*Johnius carouna*, *Nibea albiflora*, *Pennahia argentata*		In	QN, QB		[90]	
*Pleorchis sciaenae* Yamaguti, 1938		D			*Acanthopagrus berda*, *Nibea albiflora*, Sciaenidae gen. sp.		In	QN, HP		[90, 95]	
*Stephanostomum bicoronatum* (Stossich, 1883) Fuhrmann, 1928		D			*Johnius carouna*		In	QN, ND		[88]	
*Stephanostomum ditrematis* (Yamaguti, 1939) Manter, 1947		D			*Scomberoides lysan*, *Seriola dumerili*, *Seriolina nigrofasciata*		In	SCS, QN, ND		[49, 113]	
*Stephanostomum fistulariae* (Yamaguti, 1940) Manter & Van Cleave, 1951		D			*Fistularia petimba*, *Harpadon nehereus*		In	GOT, HP		[90, 95]	
*Stephanostomum hispidum* (Yamaguti, 1934) Manter, 1940		D			*Seriolina nigrofasciata*		In	SCS		[113]	
*Stephanostomum imparispine* (Linton, 1905) Manter, 1940		D			*Abalistes stellaris*, *Aluterus monoceros*, *Echeneis naucrates*, *Psettodes erumei*, *Rachycentron canadum*, *Seriolina nigrofasciata*, *Triacanthus biaculeatus*		Bc, Gi, In	GOT, GOTh, SCS		[113, 114, 119]	
*Stephanostomum orientale* (Srivastava, 1939) Madhavi, 1976		D			*Seriola dumerili*, *Seriolina nigrofasciata*		In	SCS		[113]	
*Stephanostomum tenue* (Linton, 1898) Linton, 1934		D			*Chirocentrus dorab*		S	GOT		[84]	
*Stephanostomum* sp.		D			*Ephippus orbis*, *Epinephelus coioides*, Sciaenidae gen. sp.		In	GOT		[81, 114, 133]	
*Tormopsolus carangi* Parukhin, 1976		D			*Carangoides malabaricus*		In, S	SCS		[118]	
*Tormopsolus filiformis* Sogandares-Bernal & Hutton, 1958		D			*Carangoides malabaricus*, *Rachycentron canadum*		In	GOT, SCS		[113, 119]	
*Tormopsolus orientalis* Yamaguti, 1934		D			*Carangoides malabaricus*		S	GOT, SCS		[71, 73, 113]	
**Superfamily: Opecoelioidea Ozaki, 1925**											
**Family: Opecoelidae Ozaki, 1925**											
*Allopodocotyle epinepheli* (Yamaguti, 1942) Pritchard, 1966		D			*Drepane punctata*		In	GOT		[81]	
*Allopodocotyle* sp.		D			*Epinephelus coioides*		In	QN, HP		[133]	
*Cainocreadium labracis* (Dujardin, 1845) Nicoll, 1909		D			*Scorpaenopsis cacopsis*, *Synodus variegatus*		S	KH		[27]	
*Coitocaecum gymnophallum* Nicoll, 1915		D			*Acanthopagrus berda*, *A. latus*		In	QN, HP		[90], Present study	
*Helicometra fasciata* (Rudolphi, 1819) Odhner, 1902		D			*Epinephelus bleekeri*, *E. coioides*, *E. malabaricus*, *E. quoyanus*, *E. sexfasciatus*		In	GOT, KH		[34, 90, 139]	
*Helicometra pisodonophi* Nguyen Van Ha, 2012^3^		D			*Pisodonophis cancrivorus*		In	QN, HP		[48]	
*Helicometrina nimia* Linton, 1910		D			*Epinephelus sexfasciatus*		In	QB			
*Helicometra* sp.		D			*Acanthopagrus latus*, *Epinephelus coioides*		In	QN		[133]; Present study	
*Macvicaria* sp.		D			*Amphiprion clarckii*		In	KH		[142]	
*Neonotoporus decapteri* Parukhin, 1966		D			*Decapterus* sp.		In	SCS, GOT		[112, 113]	
*Opegaster brevifistula* Ozaki, 1928		D			*Sillago sihama*		In	GOT		[90]	
*Opecoelus haduyngoi* Nguyen Van Ha, 2012		D			*Acanthopagrus berda*		In	QN, HP		[48]	
*Opecoelus pteroisi* Shen, 1986		D			*Pennahia argentata*		In	QN, HP		[88, 90]	
*Opecoelus sphaericus* Ozaki, 1925		D			*Ephippus orbis*, *Platax orbicularis*		In	GOT		[81]	
*Opecoelus* sp.		D			*Megalaspis cordyla*		In	SCS		[113]	
*Opecoelina vixiintestina* Oshmarin, 1965		D			*Terapon theraps*		In, S	GOT		[95]	
*Opecoelina* sp.		D			*Epinephelus amblycephalus*		In	QB		[90]	
*Opegaster parapristipomatis* Yamaguti, 1934		D			*Gerres filamentosus*		In	GOT		[81]	
*Opistholebes amplicoelus* Nicoll, 1915		D			*Lagocephalus lunaris*		In	HP		[90]	
*Podocotyloides petalophallus* Yamaguti, 1934		D			*Plectorhinchus cinctus*, *Plectorhinchus* sp.		In	GOT		[81]	
*Pseudopecoeloides carangis* (Yamaguti, 1938) Yamaguti, 1940		D			*Alectis indicus*, *Megalaspis cordyla*		In	SCS		[113]	
*Pseudopecoeloides* sp.		D			*Atropus atropus*		In	HP		[90]	
*Phyllotrema* sp.		D			*Pinjalo pinjalo*		In	QB		[90]	
*Pycnadenoides pagrosomi* Yamaguti, 1938		D			Sciaenidae gen. sp.		In	GOT		[95]	
*Vesicocoelium solenophagum* Tang, Hsu, Huang & Lu, 1975		D			*Gerres limbatus*		In	QB		[90]	
**Superfamily: Gorgoderoidea Looss, 1901**											
**Family: Gorgoderidae Looss, 1899**											
*Phyllodistomum carangis* (MacCallum, 1913) Cutmore & Cribb, 2018		D			*Scomberoides lysan*		In	QN, HP, SCS		[90, 113]	
*Phyllodistomum lancea* Mamaev, 1968		D			*Auxis thazard*, *Euthynnus affinis*		In	SCS		[80]	
*Phyllodistomum notosinicum* Lebedev, 1970		D			*Scomberomorus* sp., *Rastrelliger brachysoma*		In	GOT, SCS		[73, 90]	
*Phyllodistomum parukhini* Yamaguti, 1971		D			*Rachycentron canadum*		K, Ub	GOT, SCS		[106]	
*Phyllodistomum psettodi* Parukhin, 1966		D			*Psettodes erumei*		Ub	GOT		[112]	
*Phyllodistomum strictum* Oshmarin, 1965		D			*Parastromateus niger*		In	GOT		[95]	
*Phyllodistomum* sp.		D			*Nibea albiflora*		Ub	QN, HP		[88, 90]	
*Xystretrum abalisti* Parukhin, 1963		D			*Triacanthus biaculeatus*		Ub	GOT		[119]	
**Superfamily: Microphalloidea Ward, 1901**											
**Family: Faustulidae Poche, 1926**											
*Paradiscogaster drepanei* Mamaev, 1970		D			*Drepane longimana*, *D. punctata*		In	GOT		[81]	
**Family: Microphallidae Ward, 1901**											
*Microphallus* sp.		D			*Parupeneus multifasciatus*		In	KH		[27]	
**Family: Zoogonidae Odhner, 1902**											
*Plectognathotrema ovatum* Parukhin, 1964		D			*Aluterus monoceros*		In	GOT, SCS		[107, 119]	
Digenea gen. sp.		D			*Alectis indicus*, *Alepes melanoptera*, *Atropus atropos*, *A. oreolatus*, *Atule mate*, *Carangoides chrysophrys*, *C. malabaricus*, *Caranx* sp., *Decapterus* sp., *Echeneis naucrates*, *Epinephelus coioides*, *Gnathanodon speciosus*, *Megalaspis cordyla*, *Pomadasys argenteus*, *Psettodes erumei*, *Rachycentron canadum*, *Scomberoides lysan*, *Selar crumenophthalmus*, *S. crumenophthalmus*, *Selaroides leptolepis*, *Seriola dumerili*, *Seriolina nigrofasciata*, Carangidae gen. sp.		Bc, Ve, Mu, In, S	SCS, GOT		[19, 34, 133]	
**Class: Cestoda Rudolphi, 1808**											
**Subclass: Eucestoda Southwell, 1930**											
**Order: Bothriocephalidea Kuchta, Scholz, Brabec & Bray, 2008**											
**Family: Bothriocephalidae Blanchard, 1849**											
*Bothriocephalus manubriformis* (Linton, 1889) Ariola, 1900		C			*Makaira* sp.		In	GOT		[73]	
**Order: Diphyllobothriidea Kuchta, Scholz, Brabec & Bray, 2008**											
**Family: Diphyllobothriidae Lühe, 1910**											
*Diphyllobothrium* sp.		C			*Alepes melanoptera*, *Atule mate*		In, Mu	SCS		[113]	
**Order: Lecanicephalidea Hyman, 1951**											
**Family: Cephalobothriidae Pintner, 1928**											
*Tylocephalum* sp.		C			*Epinephelus bleekeri*, *E. coioides*, *E. malabaricus*, *Lates calcarifer*		In	KH		[140]	
**Order: Onchoproteocephalidea Caira, Jensen, Waeschenbach, Olson & Littlewood, 2014**											
**Family: Proteocephalidae La Rue, 1911**											
*Proteocephalus* sp.		C			*Lates calcarifer*		In	KH		[122]	
**Order: Tetraphyllidea Carus, 1863**											
**Family: Tetraphyllidea *incertae sedis***											
*Scolex* sp.		C			*Auxis thazard*, *Thunnus* sp.		Bc, L	SCS		[80]	
Tetraphyllidea gen. sp. plerocercoid		C			*Abalistes stellaris*, *Acanthocepola limbata*, *Carangoides malabaricus*, *Caranx* sp., *Decapterus muroadsi*, *Decapterus* sp., *Drepane punctata*, *Echeneis naucrates*, *Epinephelus coioides*, *Mene maculata*, *Parastromateus niger*, *Protonibea diacanthus*, *Selar crumenophthalmus,* Carangidae gen. sp.		Bc, Gb, In, S	GOT, SCS		[19, 73, 81, 119, 133]; Present study	
**Order: Trypanorhyncha Diesing, 1863**											
**Suborder: Trypanobatoida Olson, Caira, Jensen, Overstreet, Palm & Beveridge, 2010**											
**Superfamily: Tentacularioidea Poche, 1926**											
**Family: Tentaculariidae Poche, 1926**											
*Nybelinia* sp.		C			*Alectis indicus*, *Carangoides malabaricus*, *Caranx* sp., *Cepola schlegelii*, *Decapterus muroadsi*, *Decapterus* sp., *Leiognathus* sp., *Megalaspis cordyla*, *Mene maculata*, *Parastromateus niger*, *Platax orbicularis*, *Pomadasys argenteus*, *Rastrelliger kanagurta*, *Scomberoides lysan*, *Scomberomorus commerson*, *S. guttatus*, *Scorpaenodes* sp., *Selar crumenophthalmus*, *Selaroides leptolepis*, *Selaroides* sp., *Seriola dumerili*, *Seriola* sp., *Seriolina nigrofasciata*, *Triacanthus biaculeatus,* Carangidae gen. sp., Leiognathidae gen. sp., Scombridae gen. sp.		Bc, Go, In, K, L, S	GOT, SCS		[73, 81, 119, 143]	
*Tentacularia coryphaenae* Bosc, 1802		C			*Acanthocybium solandri*		SW	HP		Present study	
**Superfamily: Eutetrarhynchoidea Guiart, 1927**											
**Family: Eutetrarhynchidae Guiart, 1927**											
*Dollfusiella* sp. A^4^		C			*Telatrygon zugei*		Sv	HP		Present study	
*Dollfusiella* sp. B		C			*Neotrygon kuhlii*		Sv	HP		Present study	
*Oncomegas wageneri* (Linton, 1890) Dollfus, 1929		C			*Acanthocepola limbata*, *Cepola schlegelii*, *Cepola* sp.		Bc	GOT		[81]	
*Prochristianella* sp.		C			*Telatrygon zugei*		Sv	HP		Present study	
Eutetrarhynchidae gen. sp.		C			*Chirocentrus dorab*, *Ilisha* sp.		Bc	GOT		[81]	
**Family: Rhinoptericolidae Carvajal & Campbell, 1975**											
*Shirleyrhynchus* cf. *butlerae* Beveridge & Campbell, 1988		C			*Neotrygon kuhlii*, *Telatrygon zugei*		Sv	HP		Present study	
**Suborder: Trypanoselachoida Olson, Caira, Jensen, Overstreet, Palm & Beveridge, 2010**											
**Superfamily: Lacistorhynchoidea Guiart, 1927**											
**Family: Pterobothriidae Pintner, 1931**											
*Pterobothrium platycephalum* (Shipley & Hornell, 1906) Dollfus, 1930		C			*Mene maculata*, *Parastromateus niger*, *Platax orbicularis*		Bc	GOT		[81]	
**Family: Lacistorhynchidae Guiart, 1937**											
*Grillotia* sp.		C			*Drepane punctata*, *Platax orbicularis*		Bc	GOT		[81]	
**Superfamily: Otobothrioidea Dollfus, 1942**											
**Family: Lacistorhynchidae Guiart 1937**											
*Callitetrarhynchus gracilis* (Rudolphi, 1819) Pintner, 1931		C			*Auxis thazard*, *Epinephelus coioides*, *E. malabaricus*, *Euthynnus affinis*, *Thunnus thynnus*, *Thunnus* sp.		S	SCS, KH		[80, 140]	
**Family: Otobothriidae Dollfus, 1942**											
*Otobothrium* sp.		C			*Abalistes stellaris*, *Aluterus monoceros*, *Auxis thazard*, *Cepola schlegelii*, *Cepola* sp., *Drepane punctata*, *Ephippus orbis*, *Gymnocranius griseus*, *Leiognathus equula*, *Leiognathus* sp., *Parastromateus niger*, *Platax orbicularis*, *Pomadasys argenteus,* Leiognathidae gen. sp.		Bc, Sw, In	GOT		[80, 81, 119]	
**Family: Pseudotobothriidae Palm, 1995**											
*Parotobothrium balli* (Southwell, 1929) Palm, 2004		C			*Epinephelus bleekeri*, *E. coioides*, *E. malabaricus*		S	KH		[135]	
Trypanorhyncha gen. sp.		C			*Abalistes stellaris*, *Acanthocepola limbata*, *Alepes melanoptera*, *Aluterus monoceros*, *Atule mate*, *Carangoides malabaricus*, *Caranx* sp., *Cepola schlegelii*, *Decapterus* sp., *Echeneis naucrates*, *Ephippus orbis*, *Gymnocranius griseus*, *Mene maculata*, *Parastromateus niger*, *Pomadasys argenteus*, *Psettodes erumei*, *Rachycentron canadum*, *Selaroides leptolepis,* Carangidae gen. sp.		Bc, Gi, In, Mu	GOT, GOTh, SCS		[19, 81, 109, 113, 115, 117, 119]	
Cestoda gen. sp.		C			*Abalistes stellaris*, *Alectis indicus*, *Alepes melanoptera*, *Atropus atropos*, *A. oreolatus*, *Atule mate*, *Carangoides chrysophrys*, *C. malabaricus*, *Caranx* sp., *Decapterus* sp., *Echeneis naucrates*, *Epinephelus coioides*, *Gnathanodon speciosus*, *Megalaspis cordyla*, *Psettodes erumei*, *Rachycentron canadum*, *Selar crumenophthalmus*, *Selaroides leptolepis*, *Seriola dumerili*, *Seriolina nigrofasciata,* Carangidae gen. sp.			GOT		[19, 109, 133]	
**Phylum: Nematoda Rudolphi, 1808**											
**Class: Chromadorea Inglis, 1983**											
**Order: Rhabditida Chitwood, 1933**											
**Suborder: Spirurina Railliet & Henry, 1915**											
**Infraorder: Ascaridomorpha De Ley & Blaxter, 2002**											
**Superfamily: Ascaridoidea Baird, 1853**											
**Family: Anisakidae Railliet & Henry, 1912**											
*Anisakis* sp. Larvae		N			*Abalistes stellaris*, *Acanthocepola limbata*, *Alectis indicus*, *Alepes melanoptera*, *Atropus atropos*, *A. oreolatus*, *Atule mate*, *Auxis thazard*, *Carangoides chrysophrys*, *Carangoides malabaricus*, *Caranx* sp., *Cepola schlegelii*, *Cepola* sp., *Chirocentrus dorab*, *Decapterus muroadsi*, *Decapterus* sp., *Drepane longimana*, *D. punctata*, *Dussumieria elopsoides*, *Echeneis naucrates*, *Ephippus orbis*, *Epinephelus bleekeri*, *E. coioides*, *E. malabaricus*, *Euthynnus affinis*, *Gerres filamentosus*, *Gerres* sp., *Gymnocranius griseus*, *Ilisha* sp., *Leiognathus equulus*, *Leiognathus* sp., *Lutjanus russellii*, *L. sebae*, *Makaira* sp., *Megalaspis cordyla*, *Mene maculata*, *Pampus argenteus*, *Parastromateus niger*, *Platax orbicularis*, *Pomadasys argenteus*, *Psettodes erumei*, *Rachycentron canadum*, *Rastrelliger kanagurta*, *Sardinella* sp., *Scomberoides lysan*, *Scomberomorus commerson*, *S. guttatus*, *Scomberomorus* sp., *Selar crumenophthalmus*, *Selaroides leptolepis*, *Selaroides* sp., *Seriola dumerili*, *Seriola* sp., *Seriolina nigrofasciata*, *Thunnus thynnus*, *Xiphias* sp., Carangidae gen. sp., Chaetodontidae gen. sp., Scombridae gen. sp., Leiognathidae gen. sp.		Bc, Sw, In,	GOT, SCS, GOTh		[19, 73, 80, 113, 118, 119, 140]	
*Contracaecum* sp.		N			*Abalistes stellaris*, *Alectis indicus*, *Alepes melanoptera*, *Atropus atropos*, *Atule mate*, *Auxis thazard*, *Carangoides malabaricus*, *Caranx* sp., *Decapterus* sp., *Echeneis naucrates*, *Euthynnus affinis*, *Lutjanus russellii*, *L. sebae*, *Makaira* sp., *Megalaspis cordyla*, *Mene maculata*, *Pampus argenteus*, *Parastromateus niger*, *Platax orbicularis*, *Psettodes erumei*, *Rachycentron canadum*, *Rastrelliger kanagurta*, *Sardinella* sp., *Scomberoides lysan*, *Scomberomorus commerson*, *S. guttatus*, *Scomberomorus* sp., *Selar crumenophthalmus*, *Selar* sp., *Selaroides leptolepis*, *Selaroides* sp., *Seriola dumerili*, *Seriola* sp., *Seriolina nigrofasciata*, *Thunnus thynnus*, *Xiphias* sp., Carangidae gen. sp., Scombridae gen. sp.		Bc, Sw, In, K	GOT, SCS, GOTh		[19, 73, 113, 118, 119, 140]	
**Family: Ascarididae Baird, 1853**											
*Porrocaecum* sp.		N			*Abalistes stellaris*, *Alectis indicus*, *Alepes melanoptera*, *Atropus atropos*, *Atule mate*, *Carangoides chrysophrys*, *C. malabaricus*, *Caranx* sp., *Chirocentrus dorab*, *Decapterus muroadsi*, *Decapterus* sp., *Dussumieria elopsoides*, *Echeneis naucrates*, *Gnathanodon speciosus*, *Ilisha* sp., *Lutjanus lutjanus*, *L. russellii*, *L. sebae*, *Megalaspis cordyla*, *Pampus argenteus*, *Psettodes erumei*, *Rastrelliger kanagurta*, *Sardinella* sp., *Scomberoides lysan*, *Scomberomorus commerson*, *S. guttatus*, *Scomberomorus* sp., *Selar crumenophthalmus*, *Selaroides leptolepis*, *Selaroides* sp., *Seriola dumerili*, *Seriola* sp., *Seriolina nigrofasciata*, *Triacanthus biaculeatus,* Carangidae gen. sp., Scombridae gen. sp.		Bc, In	GOT, GOTh, SCS		[73, 81, 109, 113, 119]	
**Family: Raphidascarididae Hartwich, 1954**											
*Hysterothylacium aduncum* (Rudolphi, 1802)		N			*Epinephelus bleekeri*, *E. coioides*, *E. malabaricus*		Sw	KH		[135]	
*Hysterothylacium chorinemi* (Parukhin, 1966) Bruce & Cannon, 1989		N			*Atule mate*, *Scomberoides lysan*		In	SCS		[113]	
*Hysterothylacium incurvum* (Rudolphi, 1819) Deardorff & Overstreet, 1980		N			*Xiphias* sp.		In	GOT		[73]	
*Hysterothylacium saba* (Yamaguti, 1941) Deardorff & Overstreet, 1980		N			Scombridae gen. sp.		In	GOT		[73]	
*Hysterothylacium* sp.		N			*Amphiprion clarckii*, *A. frenatus, A. polymnus*		L	KH		[142]	
*Iheringascaris inquies* (Linton, 1901) Deardorff & Overstreet, 1980		N			*Rachycentron canadum*		In	GOT, SCS		[118]	
*Raphidascaris* sp. (Larva)		N			*Echeneis naucrates*, *Epinephelus bleekeri*, *E. coioides*, *E. malabaricus*			SCS		[115, 135]	
**Superfamily: Seuratoidea Hall, 1916**											
**Family: Cucullanidae Cobbold, 1864**											
*Cucullanus decapteri* Parukhin, 1966		N			*Decapterus* sp.		In	SCS		[113]	
*Cucullanus heterochrous* Rudolphi, 1802		N			*Psettodes erumei*		In	SCS		[114]	
*Cucullanus* sp.		N			*Drepane punctata*, *Gymnocranius griseus*		In	GOT		[81]	
**Infraorder: Gnathostomatomorpha De Ley & Blaxter, 2002**											
**Superfamily: Gnathostomatoidea Railliet, 1895**											
**Family: Gnathostomatidae Railliet, 1895**											
*Echinocephalus spinosissimus* (von Linstow in Shipley et Hornell, 1905)		N			*Abalistes stellaris*, *Echeneis naucrates*		Io	SCS, GOT		[118, 119]	
*Echinocephalus* sp.		N			*Acanthopagrus latus*, *Echeneis naucrates*, *Ilisha* sp., *Triacanthus biaculeatus*,		In	GOT. SCS		[81, 115, 119] Present study	
**Infraorder: Spirurina incertae sedis**											
**Superfamily: Dracunculoidea Stiles, 1907**											
**Family: Philometridae Baylis & Daubney, 1926**											
*Buckleyella buckleyi* Rasheed, 1963		N			*Scomberoides lysan*		Py	SCS		[111, 118]	
*Philometra balistii* (Rasheed, 1963) Vidal-Martínez, Aguirre- Macedo & Moravec, 1995		N			*Abalistes stellaris*		Oe	GOTh, GOT		[116, 119]	
*Philometra spinosa* Vo, 2010^5^		N			*Epinephelus coioides*		Fi	KH		[135]	
*Philometra* sp.		N			*Abalistes stellaris*, *Carangoides malabaricus*, *Caranx* sp., *Decapterus* sp., *Epinephelus bleekeri*, *E. coioides*, *E. malabaricus*, *Megalaspis cordyla*, *Parastromateus niger*, *Psettodes erumei*, *Sardinella* sp., *Triacanthus biaculeatus,* Leiognathidae gen. sp		Op, Fi	GOT, SCS, QN, KH		[19, 71, 73, 117–119]	
*Philometra* sp. 1^6^		N			*Epinephelus coioides*		Fi	ND		[133]	
*Philometra* sp. 2		N			*Epinephelus coioides*		eyes	HP		[133]	
*Philometroides atropi* (Parukhin, 1966) Moravec & Ergens, 1970		N			*Atropus atropos*		Bc	GOT, SCS		[112, 113]	
*Philometroides* sp.		N			*Euthynnus affinis*, *Rachycentron canadum*		Bc	GOT		[117]	
**Infraorder: Spiruromorpha De Ley & Blaxter, 2002**											
**Superfamily: Camallanoidea Travassos, 1920**											
**Family: Camallanidae Railliet & Henry, 1915**											
*Camallanus* sp.		N			*Echeneis naucrates*, *Psettodes erumei*		In	GOT, SCS		[114, 115]	
*Procamallanus* (*Spirocamallanus*) *guttatusi* (Andrade-Salas, Pineda-López & García-Magaña, 1994)		N			*Epinephelus bleekeri*, *E. coioides*, *E. malabaricus*		In	KH		[140]	
*Procamallanus istiblenni* (Noble, 1966) Moravec & Sey, 1988		N			*Amphiprion clarckii*, *A. frenatus*, *A. perideraion*, *A. polymnus*		In	KH		[142]	
**Superfamily: Habronematoidea Ivaschkin, 1961**											
**Family: Cystidicolidae Skrjabin, 1946**											
*Ascarophis* sp.		N			*Echeneis naucrates*, *Epinephelus malabaricus*, *Gymnocranius griseus*		In	GOT, SCS		[81, 114, 115, 140]	
*Ctenascarophis gastricus* Mamaev, 1968		N			*Auxis thazard*, *Euthynnus affinis*,		In	SCS		[80]	
*Prospinitectus mollis* (Mamaev, 1968) Petter, 1979		N			*Auxis thazard*, *Euthynnus affinis*, *Thunnus thynnus*,		In	SCS		[80]	
*Spinitectus echenei* Parukhin, 1967		N			*Echeneis naucrates*		In	SCS		[115]	
*Spinitectus* sp.		N			*Auxis thazard*		In	SCS		[80]	
**Superfamily: Physalopteroidea Railliet, 1893**											
**Family: Physalopteridae Railliet, 1893**											
*Cestocephalus petterae* (Le-Van-Hoa, Pham-Ngoc-Khue & Nguyen-Thi-Lien, 1972) Moravec & Justine, 2018		N			*Polynemus plebeius*		In	SCS		[52]	
*Rasheedia deblocki* (Le-Van-Hoa, Pham-Ngoc-Khue & Nguyen-Thi-Lien, 1972) Moravec & Justine, 2018		N			*Eleutheronema tetradactylum*		In	SCS		[52]	
**Superfamily: Thelazioidea Skrjabin, 1915**											
**Family: Rhabdochonidae Skrjabin, 1946**											
*Heptochona dorabi* (Mamaev, 1968) Moravec, 1975		N			*Chirocentrus dorab*		In	GOT		[81]	
**Class: Enoplea Inglis, 1983**											
**Subclass: Dorylaimia Inglis, 1983**											
**Order: Trichinellida Hall, 1916**											
**Superfamily: Trichinelloidea Ward, 1907**											
**Family: Capillariidae Railliet, 1915**											
*Capillaria ariusi* (Parukhin, 1989) Arthur & Te, 2006		N			*Arius* sp.		In	GOTh		[119]	
*Capillaria* sp.		N			*Acanthopagrus latus*, *Epinephelus coioides*, *Gerres filamentosus*		In	GOT, SCS		[81] Present study	
*Pseudocapillaria* (*Pseudocapillaria*) *echenei* (Parukhin, 1967) Moravec, 1982		N			*Echeneis naucrates*		In	SCS		[115, 119]	
Nematoda gen. sp.		N			*Alectis indicus*, *Alepes melanoptera*, *Atropus atropos*, *A. oreolatus*, *Atule mate*, *Carangoides chrysophrys*, *C. malabaricus*, *Caranx* sp., *Chirocentrus dorab*, *Decapterus muroadsi*, *Decapterus* sp., *Echeneis naucrates*, *Gnathanodon speciosus*, *Megalaspis cordyla*, *Psettodes erumei*, *Rachycentron canadum*, *Selar crumenophthalmus*, *Selar* sp., *Selaroides leptolepis*, *Seriola dumerili*, *Seriolina nigrofasciata*, Carangidae gen. sp.			GOT, SCS		[19, 114, 119]	
**Phylum: Acanthocephala Kohlreuther, 1771**											
**Class: Eoacanthocephala Van Cleave, 1936**											
**Order: Neoechinorhynchida Ward, 1917**											
**Family: Neoechinorhynchidae Ward, 1917**											
*Neoechinorhynchus ampullata* Amin, Ha & Ha, 2011		A			*Megalops cyprinoides*		In	GOT		[4]	
*Neoechinorhynchus* (*Neoechinorhynchus*) *ascus* Amin, Ha & Ha, 2011		A			*Moolgarda seheli*		In	GOT		[4]	
*Neoechinorhynchus* (*Neoechinorhynchus*) *dimorphospinus* Amin & Sey, 1996		A			*Liza subviridis*		In	KG		[14]	
*Neoechinorhynchus* (*Neoechinorhynchus*) *johnii* Yamaguti, 1939		A			*Eleutheronema tetradactylus*, *Johnius carouna*, *Johnius* sp. *Otolithes ruber*,		In	VT, BL, KH, QN, QB		[11]	
*Neoechinorhynchus* (*Neoechinorhynchus*) *longinucleatus* Amin, Ha & Ha, 2011		A			*Strongylura strongylura*		In	GOT		[4]	
*Neoechinorhynchus* (*Neoechinorhynchus*) *manubrianus* Amin, Ha & Ha, 2011		A			*Johnius carouna*, *Nibea albiflora*		In	GOT		[4]	
*Neoechinorhynchus* (*Neoechinorhynchus*) *pennahia* Amin, Ha & Ha, 2011		A			*Pennahia argentata*		In	GOT		[4]	
*Neoechinorhynchus (Neoechinorhynchus) plaquensis* Amin, Ha & Ha, 2011		A			*Clupanodon thrissa*		In	HP		[90]	
**Order: Gyracanthocephala Van Cleave, 1936**											
**Family: Quadrigyridae Van Cleave, 1920**											
*Acanthogyrus* (*Acanthosentis*) *fusiformis* Amin, Chaudhary, Heckmann, Ha & Singh, 2019		A			*Arius* sp.		In	SCS, BL		[10]	
*Acanthogyrus* (*Acanthosentis*) *indicus* Tripathi, 1959		A			*Pomadasys argenteus*,		In	GOT		[81]	
**Class: Palaeacanthocephala Meyer, 1931**											
**Order: Echinorhynchida Southwell & Macfie, 1925**											
**Family: Arhythmacanthidae Yamaguti, 1935**											
*Heterosentis holospinus* Amin, Heckman & Ha, 2011		A			*Plotosus lineatus*		In	QN		[5]	
*Heterosentis mongcai* Amin, Heckmann & Ha, 2014		A			*Acreichthys* sp., *Epinephelus* sp.		In	QN		[7]	
*Heterosentis paraholospinus* Amin, Heckmann & Ha, 2018		A			*Leiognathus equulus*, *Megalaspis cordyla*, *Nuchequula flavaxilla*		In	BT, KH, QN		[9]	
**Family: Cavisomidae Meyer, 1932**											
*Filisoma indicum* Van Cleave, 1928		A			*Scatophagus argus*		In	KG		[7]	
*Neorhadinorhynchus atypicalis* Amin & Ha, 2011		A			*Siganus fuscescens*		In	QN		[3]	
*Neorhadinorhynchus nudum* (Harada, 1938) Yamaguti, 1939		A			*Auxis thazard*, *Decapterus* sp., *Euthynnus affinis*, *Thunnus thynnus*		In	KH, SCS		[9, 80, 119]	
**Family: Echinorhynchidae Cobbold, 1879**											
*Echinorhynchus* sp.		A			*Amphiprion clarckii*		In	KH		[142]	
**Family: Paracanthocephalidae Golvan, 1960**											
*Acanthocephalus halongensis* Amin & Ha, 2011		A			*Decapterus kurroides*		In	NA		[3]	
**Family: Rhadinorhynchidae Lühe, 1912**											
*Australorhynchus multispinosus* Amin, Heckmann & Ha, 2018		A			*Fistularia petimba*		In	KH		[12]	
*Cathayacanthus spinitruncatus* Amin, Heckmann & Ha, 2014		A			*Leiognathus equulus*, *Nuchequula flavaxilla*		In	HP, QN		[50]	
*Gorgorhynchus medius* (Linton, 1908) Chandler, 1934		A			*Selar crumenophthalmus*		Bc, Io	GOT		[118]	
*Gorgorhynchus tonkinensis* Amin & Ha, 2011		A			*Decapterus kurroides*		In	QN		[3]	
*Gorgorhynchus* sp.		A			*Abalistes stellaris*			GOT		[119]	
*Micracanthorhynchina kuwaitensis* Amin & Sey, 1996		A			*Strongylura strongylura*		In	QN		[3]	
*Rhadinorhynchus carangis* Yamaguti, 1939		A			*Carangoides malabaricus*		In	GOT, SCS		[73, 118]	
*Rhadinorhynchus circumspinus* Amin, Rubtsova & Nguyen, 2019		A			*Triacanthus biaculeatus*		In	HP		[13]	
*Rhadinorhynchus ditrematis* Yamaguti, 1939		A			*Decapterus* sp.		In	GOT, GOTh, SCS		[71, 73]	
*Rhadinorhynchus dorsoventrospinosus* Amin, Heckmann & Nguyen Van Ha, 2011		A			*Decapterus maruadsi*		In	QN		[6]	
*Rhadinorhynchus hiansi* Soota & Bhattacharya, 1981		A			*Sarda orientalis*		–	KH		[16]	
*Rhadinorhynchus johnstoni* Golvan, 1969		A			*Cypselurus hexazona*		–	QB		[7]	
*Rhadinorhynchus laterospinosus* Amin, Heckmann & Nguyen Van Ha, 2011		A			*Alectis ciliaris*, *Auxis rochei*, *A. thazard*, *Balistes* sp., *Harpadon nehereus*, *Leiognathus equulus*, *Lutjanus bitaeniatus*, *Megalaspis cordyla*, *Nuchequula flavaxilla*, *Tylosurus* sp.		In	QN, HP, BT		[6, 15]	
*Rhadinorhynchus multispinosus* Amin, Rubtsova & Ha, 2019		A			*Decapterus maruadsi*		In	HP		[13]	
*Rhadinorhynchus pacificus Amin*, Rubtsova & Ha, 2019		A			*Auxis thazard*		In	HP		[13]	
*Rhadinorhynchus pristis* (Rudolphi, 1802) Lühe, 1911		A			*Carangoides malabaricus*		In	GOT, SCS		[73, 118]	
*Rhadinorhynchus trachuri* Harada, 1935		A			*Auxis thazard*, *Megalaspis cordyla*, *Tylosurus* sp		In	KH, BT		[2]	
**Family: Transvenidae Pichelin & Cribb, 2001**											
*Pararhadinorhynchus magnus* Van Ha, Amin, Ngo & Heckmann, 2018		A			*Scatophagus argus*		In	GOT		[50]	
*Sclerocollum neorubrimaris* Amin, Heckmann & Ha, 2018		A			*Siganus guttatus*		In	KH		[12]	
**Family: Isthmosacanthidae Smales, 2012**											
*Serrasentis sagittifer* (Linton, 1889) Linton, 1932		A			*Abalistes stellaris*, *Atropus atropos*, *Carangoides malabaricus*, *Echeneis naucrates*, *Euthynnus affinis*, *Gerres filamentosus*, *Gerres* sp., *Gymnocranius griseus*, *Lutjanus russellii*, *Pomadasys argenteus*, *Rachycentron cacadum*, *Sardinella* sp., *Scomberoides lysan*, *Trachurus declivis*, *Triacanthus biaculeatus*		Py, In, Bc	GOT, GOTh, SCS		[71, 73, 80, 81, 113, 118, 119]	
Acanthocephala gen. sp.		A			*Echeneis naucrates*, *Psettodes erumei*			GOT		[109]	
**Phylum: Annelida Lamarck, 1809**											
**Class: Clitellata Michaelsen, 1919**											
**Subclass: Hirudinea Savigny, 1822**											
**Order: Rhynchobdellida Blanchard, 1894**											
**Family: Piscicolidae Johnston, 1865**											
*Piscicola* sp.		H			*Lates calcarifer*		Gi, Sk	KH		[122]	
*Zeylanicobdella arugamensis* de Silva, 1963		H			*Epinephelus bleekeri*, *E. coioides*, *E. malabaricus*, *Lates calcarifer*, *Lutjanus argentimaculatus*		Sk	KH		[139]	
**Phylum: Arthropoda von Siebold, 1848**											
**Subphylum: Crustacea Brünnich, 1772**											
**Class: Hexanauplia Oakley, Wolfe, Lindgren & Zaharof, 2013**											
**Subclass: Copepoda Milne Edwards, 1840**											
**Order: Cyclopoida Burmeister, 1834**											
**Family: Ergasilidae Burmeister, 1835**											
*Ergasilus* sp.		Cr			*Epinephelus coioides*, *E. malabaricus*		Gi	KH		[139]	
**Order: Siphonostomatoida Burmeister, 1835**											
**Family: Caligidae Burmeister, 1835**											
*Abasia* sp.			Cr			*Saurida tumbil*	Gi		GOT		[61]
*Anuretes branchialis* Rangnekar, 1953			Cr			*Sarda* sp.	–		–		[61]
*Caligus arii* Bassett-Smith, 1898			Cr			*Arius sp*	–		–		[60]
*Caligus bonito* Wilson, 1905			Cr			*Euthynnus affinis*	–		–		[61]
*Caligus confusus* Pillai, 1961			Cr			*Abalistes stellatus*, *Decapterus* sp.	Gi		GOT		[61]
*Caligus constrictus Heller, 1865*			Cr			*Decapterus* sp.	Gi		GOT		[61]
*Caligus epidemicus* Hewitt, 1971			Cr			*Epinephelus bleekeri*, *E. coioides*, *E. malabaricus*, *E. tauvina*, *Lates calcarifer*	Sk		KH		[122, 136]
*Caligus fortis* Kabata, 1965			Cr			*Abalistes stellatus*	–		–		[61]
*Caligus multispinosus* Shen, 1957			Cr			*Pampus argenteus*	–		–		[61]
*Caligus pelamydis* Krøyer, 1863			Cr			*Sphyraena jello*	–		–		[61]
*Caligus robustus* Bassett-Smith, 1898			Cr			*Decapterus* sp.	Gi		GOT		[61]
*Caligus stromatei* Krøyer, 1863			Cr			*Epinephelus bleekeri*, *E. coioides*, *E. malabaricus*, *E. tauvina*, *Lates calcarifer*	Gi		KH		[122, 136]
*Caligus* sp.			Cr			*Acanthopagrus latus*, *Epinephelus bleekeri*, *E. coioides*	Gi		KH, QN		[133, 140] Present study
*Caligodes laciniatus* (Krøyer, 1863)			Cr			*Ablennes hians*	–		–		[61]
*Hermilius pyriventris* Heller, 1865			Cr			Unidentified Marine catfish	–		–		[61]
*Mappates plataxus* Rangnekar, 1958			Cr			*Platax teira*, *Sarda* sp.	–		–		[61]
*Lepeophtheirus atypicus* Lin, Ho & Chen, 1996			Cr			*Siganus fuscescens*	–		–		[61]
*Lepeophtheirus longipalpus* Bassett-Smith, 1898			Cr			*Arius acutirostris*	–		–		[61]
*Lepeophtheirus* sp.			Cr			Epinephelus tauvina*	Sk		GOT		[34]
*Parapetalus hirsutus* (Bassett-Smith, 1898)			Cr			*Eleutheronema tetradactylum*	–		–		[61]
*Parapetalus occidentalis* Wilson, 1908			Cr			*Sphyraena jello*	–		–		[61]
*Sinocaligus formicoides* (Redkar, Rangnekar et Murti, 1949)			Cr			*Dussumieria elopsoides*	–		–		[61]
*Synestius caliginus* Steenstrup et Lutken, 1861			Cr			*Parastromateus niger*	–		–		[61]
*Parapetalus* sp.			Cr			*Rachycentron canadum*	Gi		KH		[53]
**Family: Hatschekiidae Kabata, 1979**											
*Hatschekia foliolata* Redkar, Rangnekar & Murti, 1950			Cr			*Parastromateus niger*, *Nemipterus peronii*					[61]
*Hatschekia hanguyenvani* Kovaleva, Nguyen et Ngo, 2017			Cr				Gi		GOT		[62]
*Hatschekia rotundigenitalis* Yamaguti, 1939			Cr			Unidentified marine host	–		–		[61]
*Hatschekia* sp.			Cr			*Amphiprion polymnus*	Gi		KH		[142]
*Pseudocongericola* sp.			Cr			*Congresox talabonoides*	–		–		[61]
**Family: Kroyeriidae Kabata, 1979**											
*Kroyeria spatulata* Pearse, 1948			Cr			*Carcharhinus sorrah*	–		–		[61]
**Family: Lernaeopodidae Milne Edwards, 1840**											
*Charopinopsis quaternia* (Wilson C.B., 1935)			Cr			*Scomberoides lysan*	–		–		[61]
*Parabrachiella trichiuri* (Yamaguti, 1939)			Cr			*Lutjanus erythropterus*	Gi		GOT		[61]
*Neobrachiella* sp.			Cr			*Valamugil engeli*	–		–		[61]
*Naobranchia* sp.						*Gerres filamentosus*	–		–		[61]
**Family: Lernanthropidae Kabata, 1979**											
*Chauvanium chauvani* Kazatchenko, Kovaleva, Nguyen and Ngo, 2017			Cr			*Alepes melanoptera*	Gi		GOT		[61]
*Lernanthropus alatus* Pillai, 1964			Cr			*Alepes melanoptera*, *Caranx* sp.*, Decapterus* sp.	–		–		[61]
*Lernanthropus carangis* Pillai, 1964			Cr			*Upeneus sulfureus*	–		–		[61]
*Lernanthropus chirocentrosus* Tripathi, 1959			Cr			*Chirocentrus dorab*	–		–		[61]
*Lernanthropus cornutus* Kirtisinghe, 1937			Cr			*Ablennes hians*	–		–		[61]
*Lernanthropus francai* Nunes-Ruivo, 1962			Cr			*Larimichthys croceus*	–		–		[61]
*Lernanthropus latis* Yamaguti, 1954			Cr			*Lates calcarifer*	Gi		KH		[140]
*Lernanthropus lappaceus* Wilson, 1912			Cr			*Eleutheronema tetradactylum*, *Arius maculatus*	–		–		[61]
*Lernanthropus opisthopteri* Pillai, 1964			Cr			*Ilisha elongata*	–		–		[61]
*Lernanthropus otolithi* Pillai, 1963			Cr			*Johnius carouna*	–		–		[61]
*Lernanthropus polynemi* Richiardi, 1881			Cr			Unidentified host	–		–		[61]
*Lernanthropus sanguineus* Song, 1976.			Cr			*Lutjanus johnii*	–		–		[61]
*Lernanthropus trifoliatus* Bassett-Smith, 1898			Cr			*Arius maculatus*, *Arius* sp*.*, *Polydactylus sextarius*	–		–		[61]
*Lernanthropus villiersi* Delamare-Deboutteville et Nunes-Ruivo, 1954			Cr			*Gerres filamentosus*	–		–		[61]
*Lernanthropinus decapteri* (Pillai, 1964) Pillai, 1967			Cr			*Decapterus maruadsi*, *Elisha filigera*	–		–		[61]
*Lernanthropinus gibbosus* (Pillai, 1964)			Cr			*Saurida tumbil*	–		–		[61]
*Lernanthropinus sphyraenae* (Yamaguti et Yamasu, 1959)			Cr			*Mene maculata*	–		–		[61]
*Sagum sanguineus* (Song, 1976)			Cr			*Lutjanus johnii*	–		–		[61]
*Sagum vietnamiensis* Kazatchenko, Kovaleva, Nguyen and Ngo, 2017			Cr			–	Gi		GOT		[61]
**Family: Pseudocycnidae Wilson C.B., 1922**											
*Cybicola armatus* (Bassett-Smith, 1898) Kensley et Grindley, 1973			Cr			*Sphyraena jello*, *Euthynnus alleteratus*, S*comberomorus commerson*	–		–		[61]
*Pseudocycnus appendiculatus* Heller, 1865			Cr			*Auxis thazard*, *Euthynnus affinis, E. alleteratus*	–		–		[61]
**Family: Trebiidae Wilson C.B., 1905**											
*Trebius elongatus* Capart, 1953			Cr			*Taeniura meyeni*	–		–		[61]
**Class: Malacostraca Latreille, 1802**											
**Order: Isopoda Latreille, 1817**											
**Family: Corallanidae Hansen, 1890**											
*Alcirona* sp.		Cr			*Epinephelus coioides*		Sk	QN, HP		[133]	
*Corallana* sp.		Cr			Epinephelus tauvina*		Sk	GOT		[34]	
**Family: Cymothoidae Leach, 1818**											
*Ceratothoa verrucosa* (Schioedte & Meinert, 1883)		Cr			*Epinephelus coioides*		Gi	KH		[135]	
**Family: Gnathiidae Leach, 1814**											
*Gnathia* sp.		Cr			*Epinephelus bleekeri*, *E. coioides*		Gi	KH		[133, 140]	
Gnathiidae gen. sp.		Cr			*Epinephelus coioides*		Gi	GOT		[133]	

1 This species has not been registered in WoRMS and not confirmed by international experts.

2 *Prosorhynchus* sp. A & *Prosorhynchus* sp. B have not been identified, but they are distinct based on some morphological characters.

3 This species has not been confirmed by international experts.

4 *Dollfusiella* sp. A and B were not identified to species level, but it was possible to distinguish them based on morphology.

5 Temporary name: This species has not been registered in WoRMS and not confirmed by international experts.

6 *Philometra* sp. 1 and 2 were not fully identified, but it was possible to distinguish them based on morphology.

#### Abbreviations

1. *Taxon names:* A: Acanthocephala; C: Cestoda; Ci: Ciliophora; Cr: Crustacea; D: Digenea; H: Hirudinea; Mo: Monogenea; My: Myxozoa; N: Nematoda.
2. *Sites of infection:* Bc: Body cavity; Bvg: Blood vessel gill; Fi: Fin; Gb: Gall bladder; Gi: Gill; Gic: Gill cavity; Go: Gonad; He: Heart; Ht: Head tissues; In: Intestine; Io: Inner organs; Iw: Intestine wall; K: Kidney; L: Liver; Mu: Muscle; O: Oesophagus; Oe: Orbit of the eye; Op: Operculum; Phc: Pharyngeal cavity; Py: Pyloric caeca; S: Stomach; Sb: Swimming bladder; Sk: Skin; Sw: Stomach wall; Sv: Spiral valve; Ub: Urine bladder; Us: Under scales; Ve: Vitreous eye.
*Before 2005, *Epinephelus coioides* was misidentified as *E. tauvina.*

### Parasite richness

A total of 498 parasite species have been recorded from 225 marine fish in Vietnam belonging to the following taxa: Myxozoa and Ciliophora (8 each), Monogenea (117), Digenea (214), Cestoda (17), Nematoda (37), Acanthocephala (37), Hirudinea (2), and Crustacea (58), demonstrating the high diversity of the marine fish parasites inhabiting Vietnamese waters. The current average number of parasite species per fish species was 2.2, which was lower than the previously estimated species richness in Vietnamese (3.0) and German coastal waters (3.1) [19, 104]. This lower species richness appears to be due to a higher proportion of the fish community being investigated compared to 16 years ago (225 species *vs.* 82 fish species reported in 2006) so that a better prediction of the species richness can be made. It could also be explained by a decline in host species richness and population density, the major universal determinants of variations in parasite species richness [123]. However, the new figure of 2.2 is comparable to that of New Caledonia (1.9), the Indo-West Pacific (1.7), and Hawaii’s open Pacific waters (2.2) ([58, 103, 126], respectively). These regions’ similar latitudinal patterns could account for their parasite richness [28].

Our findings showed that only 225 of the total 1876 species of marine fish recorded in Vietnam [21] had been investigated for parasite species, accounting for only 12% of the entire fish community; this indicates that the parasite fauna of marine fishes in Vietnam is still poorly known.

### Parasite composition and proportion in marine fish of Vietnam

The present results showed that the proportion of marine fish parasites observed in Vietnam is similar to that previously described in Hawaiian waters ([103]; Fig. 2b). As mentioned, the proximity of the latitudinal range between Vietnam and Hawaii might be the reason for such similarities in the proportion of the parasite taxa.

**Figure 1:**
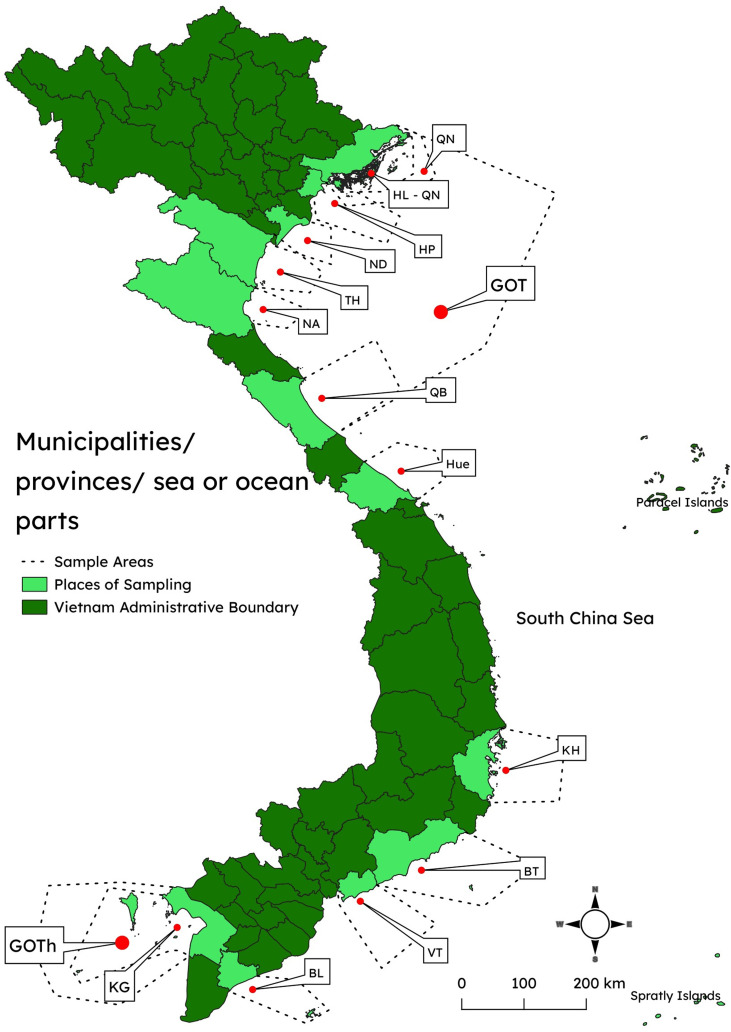
A map of Vietnam with abbreviated names of municipalities, provinces, and sea or ocean areas where samples were collected.

**Figure 2:**
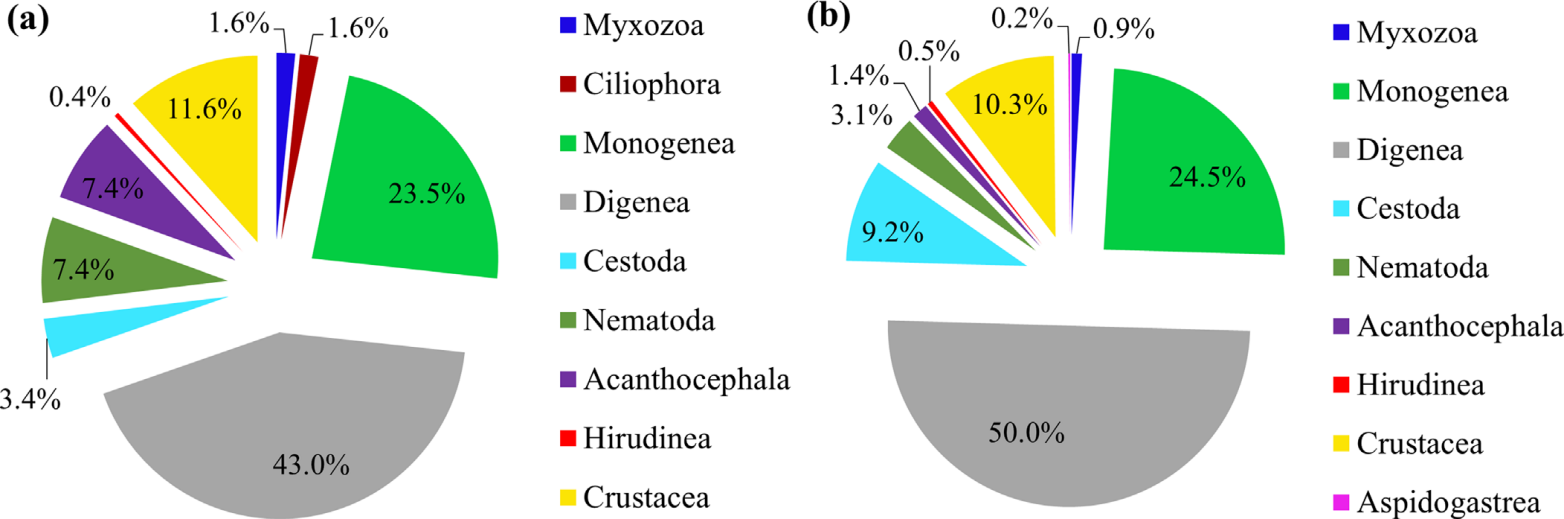
Proportion (%) of (a) the recorded fish parasite species in Vietnam (498 species, present study), and (b) fish parasites species in Hawaii (652 species [103]).

Digenea and Monogenea were the most common taxa, accounting for 43% and 23.5% of Vietnam’s total marine fish parasite fauna, respectively. The overall taxon proportions are similar to those found in Hawaii, except for Nematoda and Acanthocephala, which were higher in Vietnam than in Hawaii (7.4% and 7.4% in Vietnam *vs.* 3.1% and 1.4% in Hawaii, respectively). Many factors can influence the richness of these two parasite taxa, e.g., the zooplankton (e.g., Copepoda, Amphipoda), which may act as intermediate hosts for Nematoda and Acanthocephala and were reported to be more diverse and abundant in Vietnam than in Hawaii [56, 87]. Furthermore, since acanthocephalans use amphipods or ostracods more frequently as intermediate hosts, they are abundant in shallow waters in Ha Long Bay but not on the Hawaiian coast [56, 87]; these parasites are typically found in benthic and benthopelagic fish final hosts [54]. As a result, when compared to the 2006 list, 29 additional acanthocephalans have been added to the current local list [3–13, 50], 22 of which are new parasite species that have never been recorded anywhere other than Vietnam. Those 22 species are *Acanthocephalus halongensis* Amin & Ha, 2011; *Acanthogyrus* (*Acanthosentis*) *fusiformis* Amin, Chaudhary, Heckmann, Ha & Singh, 2019; *Neoechinorhynchus ampullata* Amin, Ha & Ha. 2011; *Neoechinorhynchus ascus* Amin, Ha & Ha, 2011; *Neoechinorhynchus longinucleatus* Amin, Ha & Ha, 2011; *Neoechinorhynchus manubrianus* Amin, Ha & Ha, 2011; *Neoechinorhynchus pennahia* Amin, Ha & Ha, 2011; *Neoechinorhynchus plaquensis* Amin, Ha & Ha, 2011; *Heterosentis holospinus* Amin, Heckmann & Ha, 2011; *Heterosentis mongcai* Amin, Heckmann & Ha, 2014; *Heterosentis paraholospinu*s Amin, Heckmann & Ha, 2018; *Australorhynchus multispinosus* Amin, Heckmann & Ha, 2018; *Cathayacanthus spinitruncatus* Amin, Heckmann & Ha, 2014; *Rhadinorhynchus circumspinus* Amin, Rubtsova & Nguyen, 2019; *Rhadinorhynchus dorsoventrospinosus* Amin, Heckmann & Ha, 2011; *Rhadinorhynchus laterospinosus* Amin, Heckmann & Ha, 2011; *Rhadinorhynchus pacificus* Amin, Rubtsova & Ha, 2019; *Rhadinorhynchus multispinosus* Amin, Rubtsova & Ha, 2019; *Neorhadinorhynchus atypicalis* Amin & Ha, 2011, *Pararhadinorhynchus magnus* Ha et al., 2018; *Gorgorhynchus tonkinensis* Amin & Ha, 2011, and *Sclerocollum neorubrimaris* Amin, Heckmann & Ha, 2018. These findings suggest that prior to 2006, acanthocephalans were neglected in the parasitological studies regarding marine fish from Vietnam.

To date, it is known that the Cestoda, particularly the orders Trypanorhyncha and Tetraphyllidea, are found in a wide range of marine fish species worldwide [101, 103]. They are tapeworms from elasmobranchs that have been considered excellent indicators of host ecology [105]. In Vietnam, cestodes accounted for only 3.4% of the total parasite fauna in marine fish (17 out of 498 species; Fig. 2a), a much lower proportion than in Hawaii (9.2% represented by 60 species; [103]; Fig. 2b); this could be because of a lack of definitive hosts (elasmobranchs) due to shallow waters or, more likely, a lack of research interest in these parasites in Vietnam.

The hirudinean parasites were in very low proportion compared to other parasite taxa. These annelids have one of the largest body sizes among the groups of parasites in marine fish and are well known to be pathogenic in farmed finfish [40]. Moreover, hirudineans occur mainly in freshwater [134] which may explain their low proportion of occurrence in marine fish observed here (0.4%; Fig. 2a). This low proportion was also observed by Palm and Bray [103] in Hawaiian waters (0.5%; Fig. 2b).

In comparison to data from Hawaii [103], Crustacea were found in similar proportions in Vietnamese marine fish (11.6% *vs.* 10.3%; Fig. 2a), but Myxozoa proportions were higher (1.6% *vs.* 0.9%), and no Ciliophora were found in Hawaii, while there was 1.6% in Vietnam. However, myxozoans have recently been reported in a low proportion in Vietnamese marine waters (0.09% equal to 2 species) when compared to other groups. However, Mackenzie and Kalavati [79] reported 223 myxosporean parasites from North Pacific marine fish, identifying them as one of the most common parasite groups in marine fish [79]. Thus, the true diversity of myxozoans in Vietnam is expected to be much higher than the current findings. The lack of information and data for this group could be attributed to rudimentary sampling methods and a lack of well-trained personnel to investigate this parasite group. Similarly, the Ciliophora, a worldwide distributed parasite of aquatic teleosts, was only found in small numbers. Depending on their host species, this taxon can range from harmless ectocommensals to dangerous parasites in fish aquaculture [79]. Bui [34] and Phan [121] reported Ciliophora as parasites causing disease in Vietnam’s finfish mariculture. These parasites that infected marine fish in Vietnam were most likely reported as multi-species groups (e.g., *Trichodina* spp.), making identification difficult. This means that the actual number of ciliates in Vietnamese marine waters may be much higher than the eight species found so far.

Parasitic crustacean species accounted for 11.6% of total marine fish parasites reported in Vietnam thus far, slightly higher than the figure found in Hawaiian marine waters (10.3%; Fig. 2). This parasite group has been identified as the most diverse and widespread subphylum of arthropods in marine fish [29, 126]. They have also been identified as critical pathogenic agents of cultured marine fish in the Asian-Pacific region [77]. Given the critical role that disease control plays in ensuring the success of aquaculture, further research on parasites is both necessary and warranted.

### Parasite composition and proportion in the Gulf of Tonkin

**Figure 3:**
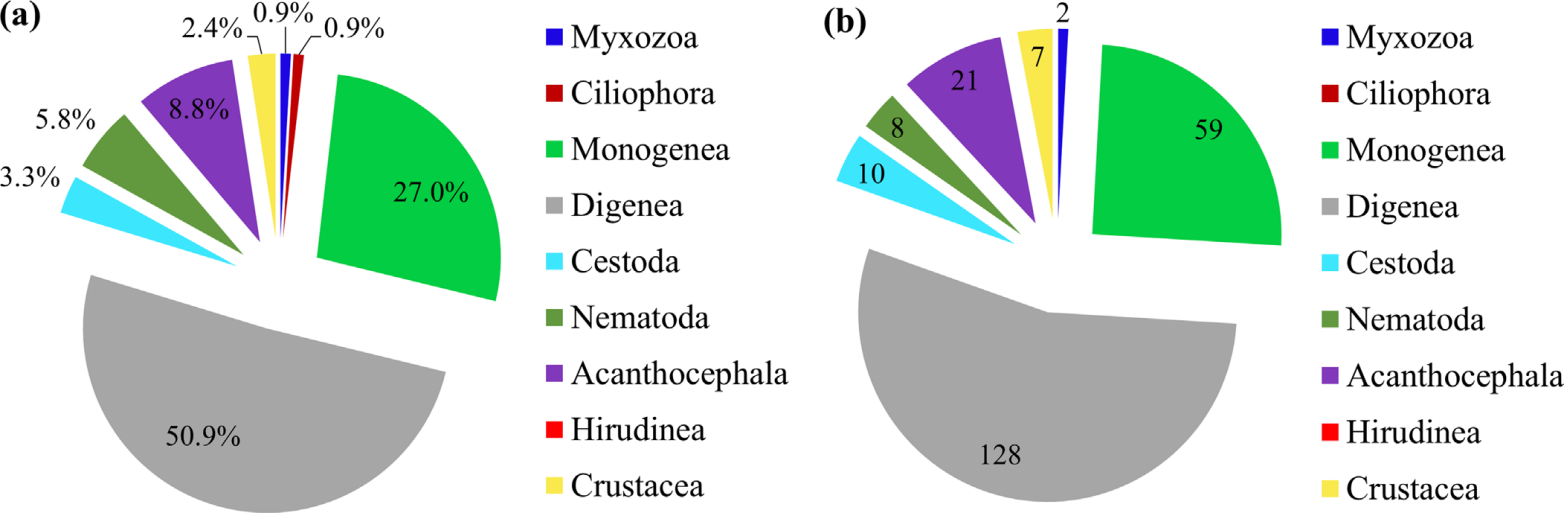
Marine fish parasites in the Gulf of Tonkin: (a) Proportion of the total recorded fish parasite species (%), and (b) Composition (species richness) of parasite species only found in the Gulf of Tonkin.

The current study presents 330 marine fish parasite species in the GOT. They are classified into eight taxa, Myxozoa (3), Ciliophora (3), Monogenea (89), Digenea (168), Cestoda (11), Nematoda (19), Acanthocephala (29), and Crustacea (8), demonstrating a high number and rich species composition in the GOT. The parasite richness in the GOT may be influenced by several factors, including geographical latitude, the environment, and the availability of intermediate hosts [55, 85]. The GOT is in South-East Asia, a region with a diverse range of parasites and hosts [125, 126], including the first intermediate host, mollusks [91], which may be the primary factor affecting endoparasite diversity, such as the digeneans (168 species) we discovered here. However, the GOT’s shallow water depth and muddy sediment limit the distribution of open-water elasmobranchs (e.g., sharks and rays), the expected final hosts of marine Cestoda, particularly Trypanorhyncha (Fig. 3). As a result, only seven Trypanorhyncha species have been identified in the GOT so far.

Most of the recorded marine parasite species in Vietnam (66.3%) have been found in the GOT, indicating that the GOT is a hotspot for interested collectors and institutions supporting research due to its high parasite biodiversity. However, apart from its natural biodiversity, most parasitological research may be concentrated in the GOT due to the urgency of fish disease research serving the high-density aquaculture area in the GOT or because conducting research here is more convenient than in other regions. The composition related to the parasite fauna in the GOT was generally similar to that of all Vietnamese marine environments (Figs. 2a and 3a). Digenea were proportionally the taxon in the GOT with the highest species richness (51%; Fig. 3a). This proportion in the GOT was higher than that observed for the whole of Vietnam (this study; Fig. 2a) and similar to that reported in Hawaii ([103], Fig. 2b). A possible explanation for the previous results relates to the environmental features of the GOT, i.e., muddy and shallow waters with rocky and limestone substrates [89, 120] which support an abundant fauna of mollusks that act as intermediate hosts for digenetic trematodes. This is also consistent with the findings of Sujatha and Madhavi [128] and Bray et al. [31], which indicated that the Digenea were typically shallow-water parasites.

Other major parasite groups in the GOT reported in this study (Fig. 3a) include Monogenea (27%), Acanthocephala (8.8%), Nematoda (5.8%), Cestoda (3.3%), Crustacea (2.4%), Ciliophora (0.9%), and Myxozoa (0.9%). It is worth noting that, of the 330 species of marine fish parasites discovered in the GOT, up to 235 species (71%) are found only here and nowhere else in Vietnam (Fig. 3b). For example, the current effort revealed that up to 21/29 acanthocephalans are found only in the GOT (Fig. 3b), suggesting that they are GOT native species.

The parasite-host ratio of 2.7 was calculated based on a total of 330 parasite species that have been recorded in 122 marine fish species in the GOT. This ratio is higher than the average for Vietnamese marine water (2.2), Hawaii (2.2), New Caledonia (1.9), and the Indo–West Pacific (1.7) [58, 103, 126]. Since the GOT is thought to have around 928 marine fish species [144], only a tiny portion of the fish species has been examined for parasites (13.1%), indicating that more attention needs to be paid to exploring the region’s high diversity of marine fish parasites.

## Conclusions

This is a comprehensive review to understand the diversity and richness of Vietnam’s marine parasite fauna. In 225 fish species, 498 marine fish parasite species have been identified, compared to 247 parasites in 82 fish species in 2006. Digenea (43%) and Monogenea (23.5%) had the highest levels of species richness. According to the data currently available, most parasites in Vietnamese fish come from the GOT (330 species, equivalent to 66.3%).

In Vietnam, the estimated marine parasite richness decreased from 3.0 in 2006 to 2.2 in 2022. Only 12% of the marine fish species have been studied for parasites, so a large part of the fish community still needs to be studied to evaluate and predict better parasite richness and diversity in this tropical-subtropical country. This compilation of parasite records shows significant progress in studying marine fish parasites in Vietnam over the last 16 years. However, it is still in its infancy, leaving a sizable task for the future, as species classification is the first critical step in characterizing any ecosystem. It is a challenging but fascinating task to learn about evolutionary biology and the history of nature, while discovering the true diversity of these marine ecosystems. Understanding pathogenic threats is critical for Vietnam’s growing finfish mariculture industry. Recent research shows that parasites can be used to study climate change and the environment. Thus, studying marine fish parasites in Vietnam is urgently needed in the future, especially using molecular data to characterize and classify the fauna.

The findings of this study will help create a database for marine fish parasites in Vietnam. Additionally, it promotes aquaculture success in Vietnam, reduces the risk of fish disease transmission between countries, and reduces the risk of consuming parasite-infected fishery and aquaculture products in Vietnam and elsewhere.

## References

[1] Amin OM. 1998. Marine flora and fauna of the eastern United States: Acanthocephala. NOAA Technical Reports, 135, 1–27.

[2] Amin OM. 2020. Redescription of *Rhadinorhynchus trachuri* Harada, 1935 (Acanthocephala: Rhadinorhynchidae) from marine fish in Vietnam and California with a discussion of its zoogeography. Acta Parasitologica, 65, 77–89. 31586284 10.2478/s11686-019-00130-z

[3] Amin OM, Nguyen VH. 2011. On four species of echinorhynchid acanthocephalans from marine fish in Halong Bay, Vietnam, including the description of three new species and a key to the species of *Gorgorhynchus*. Parasitology Research, 109, 841–847. 21400117 10.1007/s00436-011-2310-y

[4] Amin OM, Ha NV, Ha DN. 2011. First report of *Neoechinorhynchus* (Acanthocephala: Neoechinorhynchidae) from marine fish of the eastern seaboard of Vietnam, with the description of six new species. Parasite, 18, 21–34. 21395202 10.1051/parasite/2011181021 PMC3671399

[5] Amin OM, Heckmann R, Ha NV. 2011. Description of two new species of *Rhadinorhynchus* (Acanthocephala, Rhadinorhynchidae) from marine fish in Halong Bay, Vietnam, with a key to species. Acta Parasitologica, 56, 67–77.

[6] Amin OM, Heckmann RA, Ha NV. 2011. Description of *Heterosentis holospinus* n. sp. (Acanthocephala: Arhythmacanthidae) from the striped eel catfish, *Plotosus lineatus*, in Halong Bay, Vietnam, with a key to species of Heterosentis and reconsideration of the subfamilies of Arhythmacanthidae. Comparative Parasitology, 78, 29–38.

[7] Amin OM, Heckmann RA, Ha NV. 2014. Acanthocephalans from fishes and amphibians in Vietnam, with descriptions of five new species. Parasite, 21, 53. 25331738 10.1051/parasite/2014052 PMC4204126

[8] Amin OM, Heckmann RA, Ha NV. 2018. Descriptions of *Acanthocephalus parallelcementglandatus* (Echinorhynchidae) and *Neoechinorhynchus (N.) pennahia* (Neoechinorhynchidae) (Acanthocephala) from amphibians and fish in Central and Pacific coast of Vietnam, with notes on *N. (N.) longnucleatus*. Acta Parasitologica, 63, 572–585. 29975656 10.1515/ap-2018-0066

[9] Amin OM, Heckmann RA, Ha NV. 2018. Descriptions of *Neorhadinorhynchus nudum* (Cavisomidae) and *Heterosentis paraholospinus* n. sp. (Arhythmacanthidae) (Acanthocephala) from fish along the Pacific coast of Vietnam, with notes on biogeography. Journal of Parasitology, 104, 486–495. 29846128 10.1645/17-176

[10] Amin OM, Chaudhary A, Heckmann RA, Ha NV, Singh HS. 2019. The morphological and molecular description of *Acanthogyrus (Acanthosentis) fusiformis* n. sp. (Acanthocephala: Quadrigyridae) from the Catfish *Arius* sp. (Ariidae) in the Pacific Ocean off Vietnam, with notes on zoogeography. Acta Parasitologica, 64, 779–796. 31332657 10.2478/s11686-019-00102-3

[11] Amin OM, Chaudhary A, Heckmann R, Ha NV, Singh HS. 2019. Redescription and molecular analysis of *Neoechinorhynchus (Neoechinorhynchus) johnii* Yamaguti, 1939 (Acanthocephala, Neoechinorhynchidae) from the Pacific Ocean off Vietnam. Parasite, 26, 43. 31335314 10.1051/parasite/2019041 PMC6650202

[12] Amin OM, Heckmann RA, Ha NV. 2019. Descriptions of two new acanthocephalans (Rhadinorhynchidae) from marine fish off the Pacific coast of Vietnam. Systematic Parasitology, 96, 117–129. 30523612 10.1007/s11230-018-9833-x

[13] Amin OM, Rubtsova NYu, Ha NV. 2019. Description of three new species of *Rhadinorhynchus* Lühe, 1911 (Acanthocephala: Rhadinorhynchidae) from marine fish off the Pacific coast of Vietnam. Acta Parasitologica, 64, 528–543. 31270659 10.2478/s11686-019-00092-2

[14] Amin OM, Sharifdini M, Heckmann R, Ha NV. 2019. On three species of *Neoechinorhynchus* (Acanthocephala: Neoechinorhynchidae) from the Pacific Ocean off Vietnam with the molecular description of *Neoechinorhynchus (N.) dimorphospinus* Amin and Sey, 1996. Journal of Parasitology, 105, 606. 31418649

[15] Amin OM, Heckmann RA, Dallarés S, Constenla M, Ha NV. 2019. Morphological and molecular description of *Rhadinorhynchus laterospinosus* Amin, Heckmann & Ha, 2011 (Acanthocephala, Rhadinorhynchidae) from marine fish off the Pacific coast of Vietnam. Parasite, 26, 14. 30838975 10.1051/parasite/2019015 PMC6402367

[16] Amin OM, Heckmann RA, Dallarés S, Constenla M, Ha NV. 2020. Morphological and molecular description of *Rhadinorhynchus hiansi* Soota and Bhattacharya, 1981 (Acanthocephala: Rhadinorhynchidae) from marine fish off the Pacific coast of Vietnam. Journal of Parasitology, 106, 56. 31995719

[17] Anderson RC, Chabaud AG, Willmott S. 2009. Keys to the nematode parasites of vertebrates: Archival volume. CABI: Wallingford.

[18] Arai HP, Arai MN, Margolis L, Kabata Z. 1989. Guide to the Parasites of Fishes of Canada. Part III: Acanthocephala [and] Cnidaria. Department of Fisheries and Oceans.

[19] Arthur JR, Te BQ. 2006. Checklist of the parasites of fishes of Viet Nam. FAO Fisheries Technical Paper. No. 369/2: Rome.

[20] Atopkin DM, Besprozvannykh VV, Ngo HD, Ha NV, Tang NV, Ermolenko AV, Beloded AY. 2017. Morphometric and molecular data of the two digenean species *Lasiotocus lizae* Liu, 2002 (Monorchiidae) and *Paucivitellosus vietnamensis* sp. n. (Bivesiculidae) from mullet fish in Tonkin Bay, Vietnam. Journal of Helminthology, 91, 346–355. 27329346 10.1017/S0022149X16000389

[21] Atopkin DM, Besprozvannykh VV, Yu Beloded A, Ngo HD, Ha NV, Tang NV. 2017. Phylogenetic relationships of Hemiuridae (Digenea: Hemiuroidea) with new morphometric and molecular data of *Aphanurus mugilis* Tang, 1981 (Aphanurinae) from mullet fish of Vietnam. Parasitology International, 66, 824–830. 28942052 10.1016/j.parint.2017.09.009

[22] Atopkin DM, Besprozvannykh VV, Ha DN, Nguyen VH, Nguyen VT, Chalenko KP. 2019. A new subfamily, *Pseudohaploporinae subfam*. n. (Digenea: Haploporidae), with morphometric and molecular analyses of two new species: *Pseudohaploporus vietnamensis* n. g., sp. n. and *Pseudohaploporus planilizum* n. g., sp. n. from Vietnamese mullet. Parasitology International, 69, 17–24. 30439471 10.1016/j.parint.2018.11.001

[23] Atopkin DM, Besprozvannykh VV, Ha DN, Nguyen VH, Nguyen VT. 2020. New species and new genus of Pseudohaploporinae (Digenea): *Pseudohaploporus pusitestis* sp. n. and *Parahaploporus elegantus* n. g., sp. n. (Digenea: Pseudohaploporinae) from Vietnamese mullet fish. Parasitology International, 75, 102023. 31715266 10.1016/j.parint.2019.102023

[24] Besprozvannykh VV, Atopkin DM, Ngo HD, Ermolenko AV, Ha NV, Tang NV, Beloded AYu. 2016. Morphometric and molecular analyses of two digenean species from the mullet: *Haplosplanchnus pachysomus* (Eysenhardt, 1892) from Vietnam and *Provitellotrema crenimugilis* Pan, 1984 from the Russian southern Far East. Journal of Helminthology, 90, 238–244. 25902283 10.1017/S0022149X15000280

[25] Besprozvannykh VV, Atopkin DM, Ngo HD, Ermolenko AV, Ha NV, Tang NV, Beloded AY. 2017. Morphometric and molecular analyses of two digenean species in mugilid fish: *Lecithaster mugilis* Yamaguti, 1970 from Vietnam and *L. sudzuhensis* n. sp. from southern Russian Far East. Journal of Helminthology, 91, 326–331. 27086772 10.1017/S0022149X16000201

[26] Billet A. 1898. Sur quelques distomes. Notes sur la faune du Haut-Tonkin II. Bulletin Biologique de la France et de la Belgique, 28, 283–309.

[27] Binh DT, Sang TQ, Tuan DNA. 2015. Digenean diversity of reef fishes in Khanh Hoa province, Viet Nam. Journal of Fisheries Science and Technology (Nha Trang Univeristy), S, 23–28.

[28] Bordes F, Morand S, Krasnov BR, Poulin R. 2010. Parasite diversity and latitudinal gradients in terrestrial mammals, in The Biogeography of Host-Parasite Interactions. Morand S, Krasnov BR, Editors. Oxford University Press: Oxford. p. 89–98.

[29] Boxshall G, Lester R, Grygier MJ, Hoeg JT, Glenner H, Shields JD, Lützen J. 2005. Crustacean parasites. Rohde K. CSIRO. pp. 123–169.

[30] Bray R-A, Justine J-L. 2013. Bucephalidae (Digenea) from epinephelines (Serranidae: Perciformes) from the waters off New Caledonia, including *Neidhartia lochepintade* n. sp. Parasite, 20, 56. 24351242 10.1051/parasite/2013055 PMC3867101

[31] Bray RA, Littlewood DTJ, Herniou EA, Williams B, Henderson RE. 1999. Digenean parasites of deep-sea teleosts: a review and case studies of intrageneric phylogenies. Parasitology, 119, S125–S144. 11254145 10.1017/s0031182000084687

[32] Bray RA, Gibson DI, Jones A. 2008. Keys to the Trematoda, vol. 3, CABI: Wallingford.

[33] Bruno D, Nowak B, Elliott D. 2006. Guide to the identification of fish protozoan and metazoan parasites in stained tissue sections. Diseases of Aquatic Organisms, 70, 1–36. 16875388 10.3354/dao070001

[34] Bui QT. 1998. Ký sinh trùng và bệnh trên cá song nuôi lồng ở vịnh Hạ long [Parasites and fish diseases of grouper cage culture in Ha Long Bay]. Research Institute for Aquaculture No. 1, 1–14.

[35] Bykhovskiĭ BE, Guzev AV, Nagibina LF. 1965. Monogenetic trematodes of the family Tetraonchoididae Bychowsky, 1951. Trudy Zoologiccheskogo Instituta, 35, 140–166.

[36] Chaudhary A, Amin OM, Heckmann R, Singh HS. 2020. The molecular profile of *Rhadinorhynchus dorsoventrospinosus* Amin, Heckmann, and Ha 2011 (Acanthocephala: Rhadinorhynchidae) from Vietnam. Journal of Parasitology, 106, 418. 32589731 10.1645/18-144

[37] Chinh NN, Ngo HD, Tuc VV, Itoh N, Yoshinaga T, Shirakashi S, Doanh PN. 2021. A new myxosporean species, *Henneguya lata* n. sp. (Myxozoa: Myxobolidae), from the gills of yellowfin seabream *Acanthopagrus latus* (Perciformes: Sparidae) in the Gulf of Tonkin, Vietnam. Parasitology Research, 120, 877–885. 33409633 10.1007/s00436-020-07031-5

[38] Chinh NN, Ha NV, Doanh PN, Violetta Y, Yoshinaga T, Shirakashi S, Hallett SL, Whipps CM. 2022. Morphological and molecular characterization of *Ceratomyxa binhthuanensis* n. sp. (Myxosporea: Ceratomyxidae) from the gall bladder of blacktip grouper *Epinephelus fasciatus* (Perciformes: Serranidae) in the East Sea of Vietnam. Parasitology Research, 121, 613–621. 35018489 10.1007/s00436-021-07419-x

[39] Chisholm LA, Whittington ID. 2007. Review of the Capsalinae (Monogenea: Capsalidae). Zootaxa, 1559, 1–30.

[40] Cruz-Lacierda ER, Toledo JD, Tan-Fermin JD, Burreson EM. 2000. Marine leech (*Zeylanicobdella arugamensis*) infestation in cultured orange-spotted grouper, *Epinephelus coioides*. Aquaculture, 185, 191–196.

[41] Dang BT, Levsen A, Schander C, Bristow GA. 2010. Some Haliotrema (Monogenea: Dactylogyridae) from cultured grouper (*Epinephelus* spp.) with emphasis on the phylogenetic position of *Haliotrema cromileptis*. Journal of Parasitology, 96, 30–39. 19697969 10.1645/GE-2140.1

[42] Dojiri M, Ho JS. 2013. Systematics of the Caligidae, copepods parasitic on marine fishes. Leiden, the Netherlands: Brill.

[43] Egorova TP. 1994. A taxonomic review of the subfamily Trochopodinae (Monogenoidea: Capsalidae). Parazitologiya, 28, 81–91.

[44] Egorova TP, Korotaeva VD. 1990. *Trochopus antigoniae* sp. nov. (Monogenoidea: Capsalidae) from fish in the South China Sea. Parazitologiya, 24, 442–446.

[45] Froese R, Pauly D. 2021. FishBase. Available from www.Fishbase.Org.

[46] Gibbons LM. 2010. Keys to the nematode parasites of vertebrates: supplementary volume. CABI: Wallingford.

[47] Gibson DI, Jones A, Bray RA. 2002. Keys to the Trematoda, vol. 1, CABI: Wallingford.

[48] Ha NV. 2012. The description of two new species *Helicometra pisodonophi* sp. n. and *Opecoelus haduyngoi* sp. n. (Trematoda: Opecoelidae) from marine fishes in Ha Long Bay, Vietnam. Academia Journal of Biology, 34, 133–138.

[49] Ha NV, Ngo HD. 2012. The description of *Stephanostomum* spp. (Trematoda: Acanthocolpidae) from fishes of Tonkin Bay. Tap Chi Sinh Hoc (Academia Journal of Biology), 32, 1–5.

[50] Ha NV, Amin OM, Ngo HD, Heckmann RA. 2018. Descriptions of acanthocephalans, *Cathayacanthus spinitruncatus* (Rhadinorhynchidae) male and *Pararhadinorhynchus magnus* n. sp. (Diplosentidae), from marine fish of Vietnam, with notes on *Heterosentis holospinus* (Arhythmacanthidae). Parasite, 25, 35. 30040609 10.1051/parasite/2018032 PMC6057740

[51] Hendrix SS. 1994. Marine flora and fauna of the eastern United States: Platyhelminthes: Monogenea. NOAA Technical Report. NMFS, 121, 1–106.

[52] Hoa LV, Khue PN, Lien NT. 1972. Étude de deux nouvelles espèces de nématodes du genre *Bulbocephalus* Rasheed, 1966, parasites des poissons de mer du Sud Viet-Nam (Remarques sur la sous-famille Bulbocephalinae Rasheed, 1966). Bulletin de la Société de Pathologie Exotique, 65, 313–322. 4678486

[53] Hoa DT, Hich TV, Giang NTT, van Ut P, Hue NTN. 2008. Một số bệnh thường gặp trên cá nuôi biển ở Khánh Hòa [Common diseases in cultured marine finfishes in Khanh hoa province]. Journal of Fisheries Science and Technology-Nha Trang University, 02, 16–22.

[54] Houston KA, Haedrich RL. 1986. Food habits and intestinal parasites of deep demersal fishes from the upper continental slope east of Newfoundland, northwest Atlantic Ocean. Marine Biology, 92, 563–574.

[55] Jackson CJ, Marcogliese DJ, Burt MD. 1997. Role of hyperbenthic crustaceans in the transmission of marine helminth parasites. Canadian Journal of Fisheries and Aquatic Sciences, 54, 815–820.

[56] Jivaluk J. 2001. Composition, abundance and distribution of zooplankton in the South China Sea, Area IV: Vietnamese waters, in Proceedings of the Fourth Technical Seminar on Marine Fishery Resources Survey in the South China Sea, Area IV: Vietnamese Waters, 18–20 September 2000, 77–93.

[57] Jones A, Bray RA, Gibson DI. 2005. Keys to the Trematoda, vol. 2, CABI: Wallingford.

[58] Justine JL. 2010. Parasites of coral reef fish: how much do we know? With a bibliography of fish parasites in New Caledonia. Belgian Journal of Zoology, 140, 155–190.

[59] Kabata Z, Margolis L. 1988. Guide to the parasites of fishes of Canada, Part II: Crustacea. Department of Fisheries and Oceans: Ottawa.

[60] Kazachenko VN, Kovaleva NN, Ngo HD, Ha NV, Thanh NV. 2014. Redescription of three caligid species of the genus *Caligus* Müller, 1785 (Copepoda: Caligidae), parasites of marine fish *Decapterus* sp. (Perciformes: Carangidae) from Tonkin Gulf, Vietnam. Academia Journal of Biology, 36, 1–11.

[61] Kazachenko VN, Kovaleva NN, Nguyen VT, Ngo HD. 2014. Taxonomic review of the parasitic copepod (Crustacea: Copepoda) fish in Vietnam. Scientific Journal of Dalrybvtuz, 31, 20–30.

[62] Kazachenko VN, Kovaleva NN, Nguyen VT, Ngo HD. 2017. Three new species and one new genus of parasitic copepods (Crustacea: Copepoda) from fishes of the South China Sea. Russian Journal of Marine Biology, 43, 264–269.

[63] Khalil LF. 1994. Keys to the cestode parasites of vertebrates. London, UK: The Natural History Museum.

[64] King RE. 1964. Three Hemiurid Trematodes from South Viet Nam. Transactions of the American Microscopical Society, 83, 435.

[65] Klein BM. 1958. The “dry” silver method and its proper use. Journal of Protozoology, 5, 99–103.

[66] Klimpel S, Busch MW, Kellermanns E, Kleinertz S, Palm HW. 2009. Metazoan deep-sea fish parasites. Verlag Natur & Wissenschaft: Berlin.

[67] Kongkeo H, Wayne C, Murdjani M, Bunliptanon P, Chien T. 2010. Current practices of marine finfish cage culture in China, Indonesia, Thailand and Vietnam. Aquaculture Asia Magazine, 15, 32–40.

[68] Kritsky DC, Nguyen HV, Ha ND, Heckmann RA. 2016. Revision of *Metahaliotrema* Yamaguti, 1953 (Monogenoidea: Dactylogyridae), with new and previously described species from the spotted scat *Scatophagus argus* (Linnaeus) (Perciformes: Scatophagidae) in Vietnam. Systematic Parasitology, 93, 321–335. 27095662 10.1007/s11230-015-9621-9

[69] Lebedev BI. 1968. Monogeneans of commercial fish in the Pacific basin. Family Heteraxinidae Price, 1962, in Helminths of Animals of the Pacific Ocean. Skrjabin KI, Mamaev YL, Editors. Izdatelstvo Akademii Nauk SSSR: Moscow. p. 38–45.

[70] Lebedev BI. 1968. New trematodes of pelagic fish of the order Perciformes in the Pacific Basin. Helminths of Animals in the Pacific Ocean, 56–64.

[71] Lebedev BI. 1968. Helminth fauna of carangid fish in the Pacific Ocean. Soobshcheniya Dal’nevostochnogo Filiala Im. B.L. Komarova Akademii Nauk SSSR, 26, 80–85.

[72] Lebedev BI. 1968. Monogeneans of fish of the New Zealand-Australian shelf in the South China Sea (Monogenoidea: Gastrocotylidae, Gastrocotylinae), in Helminths of Animals of the Pacific Ocean. Skryabin KI, Mamaev YL, Editors. Izdatelstvo Akademii Nauk SSSR: Moscow. p. 46–55.

[73] Lebedev BI. 1970. Helminths of epipelagic fishes of the South China Sea, in Helminths of Animals of Southeast Asia. Oshmarin PG, Mamaev YL, Lebedev BI, Editors. Izdatelstvo Akademii Nauk SSSR: Moscow. p. 191–216.

[74] Lebedev BI. 1986. *Monogenei podotryada* Gastrocotylinea: (Monogenea: Suborder Gastrocotylinea). Izdatelstvo Akademii Nauk SSSR: Leningrad.

[75] Lebedev BI, Mamaev YL. 1968. Two new species of *Cardicola* Short, 1953 (Trematoda: Sanguinicolidae) from fish in the South China Sea, in Helminths of Animals of the Pacific Ocean. Skryabin KI, Mamaev YL, Editors. Izdatelstvo Akademii Nauk SSSR: Moscow. p. 72–75.

[76] Lebedev BI, Parukhin AM, Roitman VA. 1970. Oligonchoinea (Monogenoidea), parasites of horse mackerel in the North Vietnam Bay (Gulf of Tonkin). Biologiya Morya, 20, 167–187.

[77] Leong TS, Tan Z, Enright WJ. 2006. Part 1 – The parasites: Important parasitic diseases in cultured marine fish in the Asia-Pacific region. AQUA Culture AsiaPacific Magazine, 14–16.

[78] Lom J, Dyková I. 2006. Myxozoan genera: Definition and notes on taxonomy, life-cycle terminology and pathogenic species. Folia Parasitologica, 53, 1–36. 16696428

[79] Mackenzie K, Kalavati C. 2014. Myxosporean parasites of marine fishes: their distribution in the world’s oceans. Parasitology, 141, 1709–1717. 25215526 10.1017/S0031182014001425

[80] Mamaev YL. 1968. Helminths of tunas of the South China Sea, in Helminths of Animals of the Pacific Ocean. Skryabin KI, Mamaev YL, Editors. Izdatelstvo Akademii Nauk SSSR: Moscow. p. 5–27.

[81] Mamaev YL. 1970. Helminths of some food fishes of the Gulf Tonkin, in Helminths of Animals of Southeast Asia. Shmarin PG, Mamaev YL, Lebedev BI, Editors. Izdatelstvo Akademii Nauk SSSR: Moscow. p. 127–190.

[82] Mamaev YL, Oshmarin PG. 1966. Trematodes of the family Acanthocolpidae Lühe, 1901 in herrings of the North-Vietnam Bay. Helminthologia, 7, 155–164.

[83] Mamaev YL, Parukhin AM. 1970. Monogeneans of the genus *Osphyobothrus* Yamaguti, 1958 (Monogenoidea, Diclidophoridae). Parazitologiya, 4, 305–311.

[84] Mamaev YL, Parukhin AM. 1972. New Monogenea of the family Plectanocotylidae Poche, 1926. Parazitologiya, 6, 65–74. 5086335

[85] Marcogliese DJ. 1995. The role of zooplankton in the transmission of helminth parasites to fish. Reviews in Fish Biology and Fisheries, 5, 336–371.

[86] Nachev M, Sures B. 2016. Environmental parasitology: Parasites as accumulation bioindicators in the marine environment. Journal of Sea Research, 113, 45–50.

[87] Nakamura EL. 1967. Abundance and distribution of zooplankton in Hawaiian waters, 1955–56. Michigan: U.S. Department of the Interior, Bureau of Commercial Fisheries.

[88] Nguyen VH. 2011. Thành phần loài giun sán ký sinh ở một số loài cá biển vịnh Hạ Long [The helminth parasite fauna of marine fishes in Ha Long Bay]. PhD Thesis, Institute of Ecology and Biological Resources. p. 1–141. (in Vietnamese).

[89] Nguyen NM. 2013. Tidal characteristics of the Gulf of Tonkin. PhD Thesis, Université Paul Sabatier-Toulouse III. p. 1–106.

[90] Nguyen VT. 2016. Nghiên cứu sán (Trematoda) ký sinh ở một số loài cá biển ven bờ từ Hải phòng đến Quảng Binh [Study of trematoda of some coastal fishes from Hai Phong to Quang Binh provinces, Vietnam]. PhD Thesis, Institute of Ecology and Biological Resources. p. 1–155.(in Vietnamese).

[91] Nguyen TT, Nguyen C. 2012. Marine zooplankton researches in Vietnam: An overview. Coastal Marine Science, 35, 221–226.

[92] Nguyen HM, Nguyen HV, Bui TN, Ha ND. 2016. Two new axinid species (Monogenea: Axinidae) from the Pharao flyingfish *Cypselurus naresii* (Günther) (Beloniformes: Exocoetidae) in the Gulf of Tonkin off Vietnam. Systematic Parasitology, 93, 387–394. 27095667 10.1007/s11230-016-9635-y

[93] Nguyen HV, Nguyen HM, Duy Ha N, Ngoc CN, Ngoc TB, Le SX, Tatonova Y, Greiman SE. 2020. Five monogenean species (Allodiscocotylidae, Heteromicrocotylidae, Microcotylidae) from the Pacific seabream *Acanthopagrus pacificus* (Perciformes: Sparidae) in the Gulf of Tonkin off Vietnam, with descriptions of three new species. Folia Parasitologica, 67, 1–14. 10.14411/fp.2020.028 33108762

[94] Nguyen HM, Nguyen HV, Tatonova YV. 2020. Two new species of *karavolicotyla* (Unnithan, 1957) (monogenea: Heteraxinidae): parasites of two sciaenid fishes (perciformes) from Vietnam. Raffles Bulletin of Zoology, 68, 434–440.

[95] Oshmarin PG. 1965. Materials on the trematode fauna of marine and freshwater fishes of the Democratic Republic of Viet Nam, in Parasitic Worms of Domestic and Wild Animals: Papers on Helminthology. Presented to Prof. A.A. Sobolev on the 40th Anniversary of His Scientific and Teaching Activity. Leonov AA, Mamaev YL, Oshmarin PG, Editors. p. 213–249.

[96] Oshmarin PG. 1965. Two new subfamilies of trematodes from fishes in the South China Sea. Helminthologia, 6, 99–107.

[97] Oshmarin PG, Mamaev YL. 1963. A new subfamily of trematodes with a closing apparatus in the bursa from fish in the South China Sea. Zoologicheskii Zhurnal, 42, 665–669.

[98] Oshmarin PG, Mamaev YL. 1963. New trematodes from fish in the Gulf of Tonkin. Helminthologia, 4, 357–365.

[99] Oshmarin PG, Mamaev YL, Parukhin AM. 1961. New genus and species of the trematode family Diploprocto-daeidae Ozaki, 1928. Helminthologia, 3, 254–260.

[100] Paladini G, Gustinelli A, Fioravanti ML, Hansen H, Shinn AP. 2009. The first report of *Gyrodactylus salaris* Malmberg, 1957 (Platyhelminthes, Monogenea) on Italian cultured stocks of rainbow trout (*Oncorhynchus mykiss* Walbaum). Veterinary Parasitology, 165, 290–297. 19700245 10.1016/j.vetpar.2009.07.025

[101] Palm HW. 2004. The Trypanorhyncha Diesing, 1863. Bogor Agricultural University: Bogor.

[102] Palm HW. 2011. Fish parasites as biological indicators in a changing world: Can we monitor environmental impact and climate change? Progress in Parasitology, Springer, 223–250.

[103] Palm HW, Bray RA. 2014. Marine fish parasitology in Hawaii. Hohenwarsleben: Westarp.

[104] Palm HW, Klimpel S, Bucher C. 1999. Checklist of metazoan fish parasites of German coastal waters. Institut fur Meereskunde an der Christian-Albrechts-Universitat Kiel: Kiel.

[105] Palm HW, Yulianto I, Piatkowski U. 2017. Trypanorhynch assemblages Indicate ecological and phylogenetical attributes of their elasmobranch final hosts. Fishes, 2, 8.

[106] Parukhin AM. 1963. New species of trematodes from fish of the South China Sea, in Helminths of Man, Animals and Plants and Their Control. Papers on Helminthology Presented to Academician K. I. Skryabin on His 85th Birthday. Skryabin KI, Editor. Moscow: Izdatelstvo Akad. Nauk. SSSR. p. 123–125.

[107] Parukhin AM. 1964. New species of trematodes of the families Prosogonotrematidae and Cephaloporidae from fish of North Vietnam (Tonkin) Gulf. Uchenye Zapiski Gorkovskogo Gosudarstvenogo Universiteta Imeni NI Lobachevskogo, Seriya. Biologicheskaya, 62, 22–26.

[108] Parukhin AM. 1964. New species of trematodes from marine fish of North Vietnam (Tonkin) Gulf. Uchenie Zapiski Gorkovskii Gosudarstv. Pedogog. Inst. Im. M. Lobachevskogo, Seriya. Biologicheskaya, 62, 133–140.

[109] Parukhin AM. 1964. Study of the helminth fauna of marine fish of North Vietnam (Tonkin) Gulf. Uchenye Zapiski Gorkovskogo Gosudarstvvennogo Pedogogicheskii Instituta Imeni M. Gorkogo, Seriya Biologicheskaya, 48, 133–140.

[110] Parukhin AM. 1965. A new trematode species from the marine fish, *Rachycentron canadum*, of the South China Sea. Gidrobiologicheskii Zhurnal, 5, 55–56.

[111] Parukhin AM. 1966. New species of trematodes parasitic in fish in the Gulf of Tonkin, in Helminth Fauna of Animals. Seas Southern, Delyamure SL, Editors. Naukova Dumka: Kiev. p. 97–104.

[112] Parukhin AM. 1966. Some new species of trematodes from marine fish of the Gulf of Tonkin. Zoologicheskii Zhurnal, 45, 1462–1466.

[113] Parukhin AM. 1966. Helminth fauna of carangid fish from the South China Sea, in Helminth Fauna of Animals in Southern Seas. Delyamure SL, Editor. Biologiya Morya: Kiev. p. 80–96.

[114] Parukhin AM. 1967. Study of the helminth fauna of the marine fish *Psettodes erumei* in the South China Sea. Uchenye Zapiski Gorkovskogo Gosudarstvvennogo Pedogogicheskii Instituta Imeni M. Gorkogo. Seriya Biologicheskaya. Nauk, 66, 18–23.

[115] Parukhin AM. 1967. On the helminth fauna of the fish *Echeneis naucrates* from the South China Sea. Uchenye Zapiski Gorkovskogo Gosudarstvvennogo Pedogogicheskii Instituta Imeni M. Gorkogo. Seriya Biologicheskaya. Nauk, 66, 24–32.

[116] Parukhin AM. 1969. *Nematobothrium rachycentri* n.sp. (Didymozoidae), a new trematode from fish in the Gulf of Tonkin. Chenye Zapiski Gorkovskogo Gosudarstvvennogo Pedogogicheskii Instituta Imeni M. Gorkogo. Seriya Biologicheskaya. Nauk, 29, 29–33.

[117] Parukhin AM. 1971. On the helminth fauna of marine fish of North Vietnam (Tonkin) Gulf. Uchenye Zapiski Gorkovskogo Gosudarstvvennogo Pedogogicheskii Instituta Imeni M. Gorkogo. Seriya Biologicheskaya. Nauk, 116, 16–18.

[118] Parukhin AM. 1976. Parasitic worms of food fishes in the southern seas. Kiev: Naukova Dumka. p. 184 (in Russian).

[119] Parukhin AM. 1989. Parasitic worms of bottom fishes of the southern seas. Kiev: Naukova Dumka. p. 155 (in Russian).

[120] Pham T, Nguyen L. 1997. Overview of the coastal fisheries of Vietnam, in Status and Management of Tropical Coastal Fisheries in Asia. Silvestre G, Pauly D, Editors. International Center for Living Aquatic Resources Management: Makati. p. 96–106.

[121] Phan TV. 2006. Nghiên cứu tác nhân gây bệnh thường gặp trên cá song, cá giò và đề xuất biện pháp phòng bệnh [The study of common disease pathogens in Grouper, Cobia and suggest preventation methods]. Research Institute for Aquaculture No. 1, 1, 1–106.

[122] Phan VU. 2013. Thành phần loài và thử nghiệm trị bệnh do ký sinh trùng gây ra trên cá chẽm (Lates calcarifer Bloch 1970) nuôi trị Khánh Hòa [Parasite species composition and the treatment trials for parasitic disease of seabass (Lates calcarifer Bloch 1790) cultured in Khanh Hoa]. Tạp Chí Khoa Học - Công Nghệ Thủy Sản, Đại Học Nha Trang[Journal of Sciences – Technology of Fisheries, Nha Trang University], 4, 55–60.

[123] Poulin R. 2014. Parasite biodiversity revisited: frontiers and constraints. International Journal for Parasitology, 44, 581–589. 24607559 10.1016/j.ijpara.2014.02.003

[124] Riemann F. 1988. Nematoda. Introduction to the Study of Meiofauna. Thiel H, Editor. Smithsonian Press: Washington, DC. p. 293–301.

[125] Rohde K. 1999. Latitudinal gradients in species diversity and Rapoport’s rule revisited: a review of recent work and what can parasites teach us about the causes of the gradients? Ecography, 22, 593–613.

[126] Rohde K. 2005. Marine parasitology. CSIRO Publishing.

[127] Shultz JW. 2018. A guide to the identification of the terrestrial Isopoda of Maryland, U.S.A. (Crustacea). ZooKeys, 801, 207–228. 10.3897/zookeys.801.24146 PMC6288251 30564037

[128] Sujatha K, Madhavi R. 1990. Comparison of digenean faunas of sillaginid fishes from inshore and offshore waters of Visakhapatnam Coast, Bay of Bengal. Journal of Fish Biology, 36, 693–699.

[129] Sures B, Nachev M, Selbach C, Marcogliese DJ. 2017. Parasite responses to pollution: what we know and where we go in “Environmental Parasitology”. Parasites & Vectors, 10, 65. 28166838 10.1186/s13071-017-2001-3 PMC5294906

[130] Thanh BN, Dalsgaard A, Evensen Ø, Murrell KD. 2009. Survey for fishborne zoonotic metacercariae in farmed grouper in Vietnam. Foodborne Pathogens and Disease, 6, 1037–1039. 19630515 10.1089/fpd.2008.0230

[131] Tran VD, Phinn S, Roelfsema C. 2012. Coral reef mapping in Vietnam’s Coastal waters from high-spatial resolution satellite and field survey data. Asian Journal of Geoinformatics, 12.

[132] Truong TV, Palm HW, Bui TQ, Ngo HTT, Bray RA. 2016. *Prosorhynchus* Odhner, 1905 (Digenea: Bucephalidae) from the orange-spotted grouper *Epinephelus coioides* (Hamilton, 1822) (Epinephelidae), including *Prosorhynchus tonkinensis* n. sp., from the Gulf of Tonkin, Vietnam. Zootaxa, 4170, 71–92. 27701274 10.11646/zootaxa.4170.1.3

[133] Truong TV, Neubert K, Unger P, Bui TQ, Ngo HTT, Palm HW, Kleinertz S. 2017. Assessment of *Epinephelus coioides* (Hamilton, 1822) aquaculture systems in the Gulf of Tonkin, Vietnam, by using fish parasites. Journal of Applied Ichthyology, 33, 1125–1136.

[134] Verdonschot PFM. 2015. Introduction to Annelida and the class Polychaeta. Thorp and Covich’s Freshwater Invertebrates, Elsevier, 509–528.

[135] Vo TD. 2010. Động vật ký sinh ở cá mú thuộc giống *Epinephelus* spp.[The parasites from groupers *Epinephelus* spp.]. PhD Thesis. Institute of Oceanography in Nha Trang. p. 1–227.

[136] Vo DT, Bristow GA, Nguyen DH, Vo DT. 2008. Parasitism of two species of Caligus (Copepoda: Caligidae) on wild and cultured grouper in Viet Nam. Journal of the Fisheries Society of Taiwan, 35, 1–9.

[137] Vo DT, Murrell D, Dalsgaard A, Bristow G, Nguyen DH, Bui TN, Vo DT. 2008. Prevalence of zoonotic metacercariae in two species of Grouper, *Epinephelus coioides* and *Epinephelus bleekeri*, and flathead Mullet, *Mugil cephalus*, in Vietnam. Korean Journal of Parasitology, 46, 77. 18552542 10.3347/kjp.2008.46.2.77 PMC2532609

[138] Vo DT, Bristow GA, Nguyen DH, Vo DT, Nguyen TNN, Tran TC. 2011. Digenean trematodes of cultured grouper (*Epinephelus coioides* and *E. bleekeri*) in Khanh Hoa Province, Vietnam. Diseases in Asian Aquaculture VII. Fish Health Section. Bondad-Reantaso MG, et al., Editors. Asian Fisheries Society: Selangor, Malaysia. p. 39–52.

[139] Vo DT, Bristow GA, Nguyen DH, Vo DT, Nguyen TNN, Tran TC. 2011. Digenean trematodes of cultured grouper (*Epinephelus coioides* and *E. bleekeri*) in Khanh Hoa Province, Vietnam, pp. 39–52, in Diseasesin Asian Aquaculture VII, Bondad-Reantaso MG, Jones JB, Corsin F, Aoki T, Editors. Fish Health Section, Asian Fisheries Society: Selangor, Malaysia. p. 385.

[140] Vo TD, Bristow GA, Nguyen HD, Vo TD, Nguyen NTN. 2012. The parasites of grouper and sea bass in Vietnam. Vietnam Agriculture Publishing House: Ho Chi Minh.

[141] Warren MB, Orélis-Ribeiro R, Ruiz CF, Dang BT, Arias CR, Bullard SA. 2017. Endocarditis associated with blood fluke infections (Digenea: Aporocotylidae: *Psettarium* cf. *anthicum*) among aquacultured cobia (*Rachycentron canadum*) from Nha Trang Bay, Vietnam. Aquaculture, 468, 549–557.

[142] Zhokhov AE, Thi HV, Kieu OLT, Pugacheva MN, Hai TNT. 2019. Parasites of anemonefish (Pomacentridae, Amphiprioninae) in the Gulf of Nha Trang, South China Sea, Vietnam. Biology Bulletin, 46, 791–803.

[143] Zhokhov AE, Pugacheva MN, Thi HV, Mikheev VN. 2020. Parasites of small cryptic coral reef fish from the South China Sea. Russian Journal of Marine Biology, 46, 88–96.

[144] Zou K. 2013. Sino-Vietnamese Fishery Agreement in the Gulf of Tonkin. Law of the Sea in East Asia, Routledge, 125–136.

